# Assessment of the genetic diversity and population structure of groundnut germplasm collections using phenotypic traits and SNP markers: Implications for drought tolerance breeding

**DOI:** 10.1371/journal.pone.0259883

**Published:** 2021-11-17

**Authors:** Seltene Abady, Hussein Shimelis, Pasupuleti Janila, Shasidhar Yaduru, Admire I. T. Shayanowako, Dnyaneshwar Deshmukh, Sunil Chaudhari, Surendra S. Manohar

**Affiliations:** 1 African Centre for Crop Improvement (ACCI), School of Agricultural, Earth and Environmental Sciences, University of KwaZulu-Natal, Scottsville, South Africa; 2 School of Plant Sciences, Haramaya University, Dire Dawa, Ethiopia; 3 International Crops Research Institute for the Semi-Arid Tropics (ICRISAT), Telangana, India; National Cheng Kung University, TAIWAN

## Abstract

Profiling the genetic composition and relationships among groundnut germplasm collections is essential for the breeding of new cultivars. The objectives of this study were to assess the genetic diversity and population structure among 100 improved groundnut genotypes using agronomic traits and high-density single nucleotide polymorphism (SNP) markers. The genotypes were evaluated for agronomic traits and drought tolerance at the International Crop Research Institute for the Semi-Arid Tropics (ICRISAT)/India across two seasons. Ninety-nine of the test genotypes were profiled with 16363 SNP markers. Pod yield per plant (PY), seed yield per plant (SY), and harvest index (HI) were significantly (p < 0.05) affected by genotype × environment interaction effects. Genotypes ICGV 07222, ICGV 06040, ICGV 01260, ICGV 15083, ICGV 10143, ICGV 03042, ICGV 06039, ICGV 14001, ICGV 11380, and ICGV 13200 ranked top in terms of pod yield under both drought-stressed and optimum conditions. PY exhibited a significant (p ≤ 0.05) correlation with SY, HI, and total biomass (TBM) under both test conditions. Based on the principal component (PC) analysis, PY, SY, HSW, shelling percentage (SHP), and HI were allocated in PC 1 and contributed to the maximum variability for yield under the two water regimes. Hence, selecting these traits could be successful for screening groundnut genotypes under drought-stressed and optimum conditions. The model-based population structure analysis grouped the studied genotypes into three sub-populations. Dendrogram for phenotypic and genotypic also grouped the studied 99 genotypes into three heterogeneous clusters. Analysis of molecular variance revealed that 98% of the total genetic variation was attributed to individuals, while only 2% of the total variance was due to variation among the subspecies. The genetic distance between the Spanish bunch and Virginia bunch types ranged from 0.11 to 0.52. The genotypes ICGV 13189, ICGV 95111, ICGV 14421, and ICGV 171007 were selected for further breeding based on their wide genetic divergence. Data presented in this study will guide groundnut cultivar development emphasizing economic traits and adaptation to water-limited agro-ecologies, including in Ethiopia.

## Introduction

Groundnut (*Arachis hypogaea* L., AABB, 2n = 4x = 40) is an important oilseed legume crop providing various products worldwide. Groundnut is a self-pollinated allotetraploid crop derived from natural hybridization involving two diploid species, *A*. *duranensis* (A genome), and *A*. *ipaensis* (B genome) followed by polyploidization [1]. Cultivated groundnut is classified into two subspecies viz. *hypogaea* (without floral axes on the main stem) and *fastigiata* (with floral axes arising from the main stem) [2]. Subspecies *hypogaea* has a spreading growth habit with side branches procumbent to decumbent and a long growth cycle. In contrast, subspecies *fastigiata* has a more erect growth habit with side branches erect to procumbent and has a shorter growth cycles [3]. There are four market types of the cultivated groundnut viz., Virginia (*A*. *hypogaea* subsp. *hypogaea* var. *hypogaea*), runner (*A*. *hypogaea subsp*. *hypogea var*. *hirstu*), Spanish (*A*.*hypogaea* subsp. *fastigiata* var. *vulgaris)*, and Valencia (*A*.*hypogaea* subsp. *fastigiata* var. *fastigaita*) [4,5]. Virginia type of groundnuts have the largest kernels and account for most of the groundnuts roasted and processed. Runners have uniform kernel sizes and are mostly used for groundnut butter. Spanish groundnuts have smaller kernels covered with reddish-brown skin and have a higher oil content than the other types of groundnuts. Valencia types of groundnuts usually have three or more small kernels in a pod and are covered in bright red skin. Valencia types are sweet that are generally preferred for fresh use as boiled groundnuts [4]. Groundnut kernels are rich sources of oil, protein, carbohydrate, minerals (e.g., P, Ca, Mg, and K), and vitamins (E, K, and B) [6]. Groundnut kernels with high oleic acid increase oil stability and confer health benefits [7]. Groundnut haulm is used for animal feed. Also, groundnut improves soil fertility through nitrogen fixation.

Drought stress associated with climate change is one of the leading constraints to groundnut production, globally threatening food production and supply [8,9]. In South Asia and sub-Saharan Africa (SSA), more than 65% and 80% of the smallholder farmers, respectively, are dependent on rain-fed crop production systems where rainfall is low and erratic [10], limiting potential production and leading to food insecurity [11,12]. In Eastern Ethiopia, where groundnut is a major legume crop, recurrent post-flowering drought stress causes low production and productivity and crop failures [13,14].

In Ethiopia, groundnut has been used for food, edible oil extraction, and animal feed. The national mean yield is 1.796 ton/ha, and the total area under groundnut production is 80,841.57 ha [15]. In the last decade, groundnut production and yield have been increased two-fold in the country [16]. Local demand for groundnut is increasing due to the emerging groundnut processing factories. Currently, smallholder farmers account for the bulk of production under rainfed conditions in the lowland and drought-prone areas of the country [17]. The yield reduction due to drought stress depends on genotype, timing, intensity, and duration [18]. Drought stress during the reproductive phase can drastically reduce groundnut yield [19]. Terminal drought can cause 33% pod yield loss in groundnut [20]. Although several introduced groundnut varieties have been released for cultivation, none are well-adapted or drought tolerant. This has rendered low production and productivity of groundnut in sub-Saharan Africa, including Ethiopia.

Breeding groundnut for drought tolerance is an effective strategy to alleviate the impact of drought stress. Groundnut improvement for drought tolerance has achieved significant milestones [21,22]. For example, ICGV 00351, a cross derivative from ICGV 87290 X ICGV 87846, was developed and released for cultivation in drought-prone areas of India [22]. Similarly, ICGV 91114, an early maturing and drought tolerant cultivar derived from a cross between ICGV 86055 x ICGV 86533 using the bulk pedigree method, was developed at ICRISAT, India. Though conventional breeding played an important role in releasing drought-tolerant groundnut varieties, the breeding progress is slow [5]. This is due to the narrow genetic base among the cultivated groundnuts [4]. The introgression of genes from wild species into the cultivated groundnut is difficult due to the ploidy differences. In addition, the adverse effects of linkage drag associated with genes from wild relatives often present a challenge to yield gain [5,23]. Yield and yield-related traits, including pod weight, shelling outturn, hundred seed weight, and the proportion of mature pods, are the most widely used traits in groundnut improvement [5,24]. Ravi et al. [25] confirmed the complex and quantitative nature of drought tolerance in groundnut. Other traits such as specific leaf area, chlorophyll content, biomass production, and harvest index have been used as surrogate traits for drought tolerance in groundnut [23,26–28].

Based on cross-compatibility, groundnut genetic resources are classified into four gene pools. The primary gene pool includes landraces, cultivars, and wild *A*. *monticola* cross-compatible with *A*. *hypogaea*. The secondary gene pool consists of diploid species from the genus *Arachis*, cross-compatible with *A*. *hypogaea*. The tertiary gene pool includes section *Procumbentes*, *which* is cross-compatible with diploid *Arachis* species. The quaternary gene pool includes *Arachis* species, partially cross-compatible with section *Arachis* [4,29]. Previous findings indicated that the groundnut’s primary gene pool could be regarded as the main source of genes for drought tolerance [5,30–32].

Profiling the genetic composition and relationships among groundnut germplasm collections is essential for breeding new cultivars. Earlier studies used phenotypic traits and marker technologies to analyze cultivated groundnut genetic diversity and population structure [2]. SSR markers have been extensively used for assessing the genetic diversity of groundnut germplasm [24,33]. For example, one hundred and forty-six polymorphic simple sequence repeat (SSR) revealed five heterotic groups among 196 groundnut cultivars [34]. However, the number of polymorphic SSR markers in groundnut remains insufficient to deploy in genetic analysis studies. Sufficient and diagnostic SSR markers are required, which is an expensive and time-bound process. [2]. Single nucleotide polymorphisms (SNP) are increasingly becoming popular markers of choice due to their high genome abundance, ease of discovery, and extremely high-throughput genotyping at a low cost per data point, with lower genotyping error rates [35–37] Studies on genetic diversity of improved groundnut germplasm are needed to aid drought tolerance breeding for Ethiopia or genetic analysis. There is a lack of information regarding the local groundnut diversity to guide the regional breeding program. Consequently, production, utilization, and improvement of the crop are highly restricted. Thus, the objectives of this study were to assess the genetic diversity and population structure among improved groundnut genotypes using phenotypic traits and high-density single nucleotide polymorphism (SNP) markers.

## Materials and methods

### Plant materials

The groundnut genetic resources were kindly supplied by the International Crops Research Institute for the Semi-Arid Tropics (ICRISAT), Patancheru, India for exclusive use for this research. The field studies were conducted using groundnut genetic resources complying with the guidelines of the Ethiopian Institute of Agricultural Research Institute (EIAR). The study evaluated 100 improved groundnut genotypes comprising diverse advanced breeding lines (Table 1). The genotypes were selected based on desirable traits, including drought tolerance, resistance to foliar diseases such as late leaf spot and rust, and kernel quality including high oil and oleic acid contents, and early-to-medium maturity. Among the test genotypes, ICGV 98412 an introduction from the International Research Institute for the Semi-Arid Tropics/India was found to be high yielding, medium maturing, and moderately resistant to late leaf spot disease [38]. This genotype was locally released in Ethiopia and referred to as Babile-1. The majority of test genotypes are recent introductions to Ethiopia and used in the groundnut breeding program. The details of the genotypes are described in Table 1.

**Table 1:** Descriptions of the groundnut genotypes used for genetic diversity analysis.

Sr. No.	Genotype	Pedigree	Trait	Origin	Market type	Sub-species	Breeding history
1	ICGV 16667	ICGV 06110 x (ICGV 06110 x Sun Oleic 95-R) F1P2-BC1F1P3-P9-P4-P5-P1-B1-B1	HOA	ICRISAT, Hyderabad	Spanish bunch	*fastigiata*	ABL
2	ICGV 93128	(ICGMS 42 x Kadiri 3) F2-B1-B2-B2-B2-B1-B1-B1	MD	ICRISAT, Hyderabad	Spanish bunch	*fastigiata*	ABL
3	ICGV 95066	(ICGV 86388 x ICGV 86029) F4-B1-B1-B2	MD	ICRISAT, Hyderabad	Spanish bunch	*fastigiata*	ABL
4	ICGV 96174	{[(Florigiant x NCAc17090) x (Dh-3-20 x PI259747)] x ICGV 88312} F2-SSD(2)-B2-B1(2)-B2-B1	MD	ICRISAT, Hyderabad	Spanish bunch	*fastigiata*	ABL
5	ICGV 97087	{(Florigiant x NCAc 17090)x[(Dh3-20 x PI259747)x ICGV 88312]} F2-SSD-SSD-B2-B1(6)	MD	ICRISAT, Hyderabad	Spanish bunch	*fastigiata*	ABL
6	ICGV 98077	[(ICGV 86185 x ICGV 86743) x Kadiri 134] F2-SSD-SSD-B1-B1-B1	MD	ICRISAT, Hyderabad	Spanish bunch	*fastigiata*	ABL
7	ICGV 01279	(ICGV 92069 x ICGV 93184) F2-SSD-B3-B1-B2-B3-B1-B1-B1-P3-B1-B1	MD	ICRISAT, Hyderabad	Spanish bunch	*fastigiata*	ABL
8	ICGV 03042	{ICGV 99160 x [ICGV 93124 x (LI x ICGS 44)]} F2-SSD-SSD-B1-B1-B1	HO	ICRISAT, Hyderabad	Spanish bunch	*fastigiata*	ABL
9	ICGV 06039	[(ICGV 92069 x ICGV 93184) x (NC Ac 343 x ICGV 86187)S23] F2-SSD-SSD-P4-P1-B1-B1	MD	ICRISAT, Hyderabad	Spanish bunch	*fastigiata*	ABL
10	ICGV 06040	[(ICGV 92069 x ICGV 93184) x (NC Ac 343 x ICGV 86187)S23] F2-SSD-SSD-P5-B1-B1-B1	MD	ICRISAT, Hyderabad	Spanish bunch	*fastigiata*	ABL
11	ICGV 07010	(ICGV 00043 x ICGV 00064) F2-SSD-SSD-P6-B1-B1-B1	MD	ICRISAT, Hyderabad	Spanish bunch	*fastigiata*	ABL
12	ICGV 10143	(ICGV 01274 x ICGV 05063) F2-SSD-SSD-P8-B1-B1	MD	ICRISAT, Hyderabad	Spanish bunch	*fastigiata*	ABL
13	ICGV 11422	(ICGV 01274 x ICGV 04124) F2-SSD-SSD-P1-B1-B1-B1-B1	MD	ICRISAT, Hyderabad	Spanish bunch	*fastigiata*	ABL
14	ICGV 11396	(ICGV 99159 x ICGV 95047) F2-SSD-SSD-P11-B1-B1-B1-B1-B1-B1-B1	MD	ICRISAT, Hyderabad	Spanish bunch	*fastigiata*	ABL
15	ICGV 11418	(ICGV 01274 x ICGV 05063) F2-SSD-SSD-P7-B1-B1-B1-B1	MD	ICRISAT, Hyderabad	Spanish bunch	*fastigiata*	ABL
16	ICGV 91223	[ICGV 87165 x (ICG 9516 x ICGS 30)] F2-B1-B1-B1-B1-B1-B1	FDR	ICRISAT, Hyderabad	Spanish bunch	*fastigiata*	ABL
17	ICGV 94118	[(J 11 x CS 52) x ICGV 86015] F2-B1-B1-B1-B2-B1-B1	FDR	ICRISAT, Hyderabad	Spanish bunch	*fastigiata*	ABL
18	ICGV 99019	(ICGV 94118 x ICGV 92209) F2-SSD(S)-B5-B1-B1	FDR	ICRISAT, Hyderabad	Spanish bunch	*fastigiata*	ABL
19	ICGV 00162	(259–2 x ICGV 93197) F2-P14-B1-B1-B2-B2-B1(SB)	FDR	ICRISAT, Hyderabad	Spanish bunch	*fastigiata*	ABL
20	ICGV 00211	(ICGV 94118 x ICGV 93388) F2-P5-B1-B1-B1-B1-B1(SB)	FDR	ICRISAT, Hyderabad	Spanish bunch	*fastigiata*	ABL
21	ICGV 00187	(ICGV 94118 x ICGV 92267) F2-P21-B1-B1-B1-B1-B1(SB)	FDR	ICRISAT, Hyderabad	Spanish bunch	*fastigiata*	ABL
22	ICGV 00213	(ICGV 94118 x ICGV 93427) F2-P23-B1-B1-B1-B1-B1(SB)	FDR	ICRISAT, Hyderabad	Spanish bunch	*fastigiata*	ABL
23	ICGV 06146	[(ICGV 92069 x ICGV 93184) x (ICGV 96246 x 92 R/75)] F2-SSD-SSD-P12-B1-B2-B1	FDR	ICRISAT, Hyderabad	Spanish bunch	*fastigiata*	ABL
24	ICGV 07120	[{[(86187x86350)x(Florix17090)]x(Dh.3-20xPI259747)} x [ICGV 87121 x ICGV 87853)xICGV 92023]] F2-B1-SSD-P8-B1-B1-B1	FDR	ICRISAT, Hyderabad	Spanish bunch	*fastigiata*	ABL
25	ICGV 10178	(ICGV 04078 x ICG 10889) F2-SSD-SSD-SSD-P1-B1-B1-B1-B1	FDR	ICRISAT, Hyderabad	Spanish bunch	*fastigiata*	ABL
26	ICGV 11380	(ICGV 07106 x ICGV 86590) F2-SSD-SSD-SSD-B1-B1	FDR	ICRISAT, Hyderabad	Spanish bunch	*fastigiata*	ABL
27	ICGV 14001	(ICGV 06142 x ICGV 07075) F2-SSD-SSD-P9-B1-B1	FDR	ICRISAT, Hyderabad	Spanish bunch	*fastigiata*	ABL
28	ICGV 14030	(ICGV 06142 x ICGV 06282) F2-SSD-SSD-P31-B1-B1	FDR	ICRISAT, Hyderabad	Spanish bunch	*fastigiata*	ABL
29	ICGV 86015	(ICGS 44 x TG 2E) F2-B1-B2-B1	EM	ICRISAT, Hyderabad	Spanish bunch	*fastigiata*	ABL
30	ICGV 93260	(ICGS 11 x ICG 4728) F2-B1-B1-B1-B1-B1A-B1-B1-B1-B1	DT	ICRISAT, Hyderabad	Spanish bunch	*fastigiata*	Cultivar
31	ICGV 93261	(ICGS 11 x ICG 4728) F2-B1-B1-B1-B1-B1A-B1-B1-B1RF-B1	DT	ICRISAT, Hyderabad	Spanish bunch	*fastigiata*	Cultivar
32	ICGV 92121	(Ah 7827 x ICGS 11) F2-B1-B1-B3-B1-B1-B1-B1-B1	DT	ICRISAT, Hyderabad	Spanish bunch	*fastigiata*	ABL
33	ICGV 99241	(ICGV 87290 x ICGV 87846) F2-P29-B1-B1-B1-B2-B1-B1-B1	DT	ICRISAT, Hyderabad	Spanish bunch	*fastigiata*	ABL
34	ICGV 00351	(ICGV 87290 x ICGV 87846) F2-P63-B1-B1-B1-B3-B1-B1-B1-B1-B1(SB)	DT	ICRISAT, Hyderabad	Spanish bunch	*fastigiata*	Cultivar
35	ICGV 01260	(ICGV 92113 x ICGV 86300) F2-P1-B1-B1-B1-B1-B1-B1-B1-B1	DT	ICRISAT, Hyderabad	Spanish bunch	*fastigiata*	ABL
36	ICGV 01265	(ICGV 94148 x ICGV 91123) F2-SSD-SSD-B4-B1-B1-B1	DT	ICRISAT, Hyderabad	Spanish bunch	*fastigiata*	ABL
37	ICGV 13200	{TAG 24-P2 x [TAG 24-P2 x (TAG 24-P2 x GPBD 4-P1_26–1)]} BC2F1P2-P11-B1-B2-B1	FDR	ICRISAT, Hyderabad	Spanish bunch	*fastigiata*	ABL
38	ICGV 07220	[(ICGV 92069 x ICGV 93184)SIL 4 x (ICGS 44 x ICGS 76)] F2-SSD-SSD-P5-B1-B1-B1	DT	ICRISAT, Hyderabad	Spanish bunch	*fastigiata*	ABL
39	ICGV 07222	[(ICGV 92069 x ICGV 93184)SIL 4 x (ICGS 44 x ICGS 76)] F2-SSD-SSD-P19-B1-B1-B1	DT	ICRISAT, Hyderabad	Spanish bunch	*fastigiata*	Cultivar
40	ICGV 13317	(ICGV 07225 x JAL 13) F2-SSD-SSD-P14-B1-B1-B1-B1	DT	ICRISAT, Hyderabad	Spanish bunch	*fastigiata*	ABL
41	ICGV 13254	(ICGV 07223 x ICGV 07405) F2-SSD-SSD-P1-B1-B1-B1-B1-B1	DT	ICRISAT, Hyderabad	Spanish bunch	*fastigiata*	ABL
42	ICGV 181026	((ICGV 06142 x Sun Oleic 95R) X Sunoleic 95-R)-P5-P1-P1-P2-P1	HOA	ICRISAT, Hyderabad	Spanish bunch	*fastigiata*	ABL
43	ICGV 15073	(ICGV 06420 × Sun Oleic 95R)F1P3-BC1F1P14-P3-P4-P9-P6-B1	HOA	ICRISAT, Hyderabad	Spanish bunch	*fastigiata*	ABL
44	ICGV 15074	(ICGV 06420 × Sun Oleic 95R)F1P3-BC1F1P14-P3-P4-P9-P8-B1	HOA	ICRISAT, Hyderabad	Spanish bunch	*fastigiata*	ABL
45	ICGV 15083	(ICGV 06420 × Sun Oleic 95R)F1P3-BC1F1P14-P3-P5-P10-P3-B1	HOA	ICRISAT, Hyderabad	Spanish bunch	*fastigiata*	Cultivar
46	ICGV 15019	(ICGV 06420 × Sun Oleic 95R)F2P191-P3-P7-B1-B1	HOA	ICRISAT, Hyderabad	Spanish bunch	*fastigiata*	ABL
47	ICGV 06420	(ICGV 87846 x ICGV 99240) F2-P1-B1-B1-B1-B1-B1-B1-B1-B3	DT	ICRISAT, Hyderabad	Spanish bunch	*fastigiata*	
48	ICGV 05155	(ICGV 99160 x ICGV 99240) F2-B3-P6-B1-B3-B2-B1-B1-B1-B1	HO	ICRISAT, Hyderabad	Spanish bunch	*fastigiata*	ABL
49	ICGV 16688	ICGV 06110 x (ICGV 06110 x Sun Oleic 95-R) F1P2-BC1F1P3-P9-P4-P32-P1-B1-B1	HOA	ICRISAT, Hyderabad	Spanish bunch	*fastigiata*	ABL
50	ICGV 03043	{ICGV 99160 x [ICGV 93124 x (LI x ICGS 44)]} F2-SSD-SSD-B3-B1-B1	HO	ICRISAT, Hyderabad	Spanish bunch	*fastigiata*	Cultivar
51	ICGV 00350	(ICGV 87290 x ICGV 87846) F2-P63-B1-B1-B1-B1-B2-B1-B1-B1-B1(SB)	DT	ICRISAT, Hyderabad	Spanish bunch	*fastigiata*	Cultivar
52	ICGV 86590	(X14-4-B-19-B x PI 259747) F2-B2-B1-B1-B1-B1-B2	FDR	ICRISAT, Hyderabad	Spanish bunch	*fastigiata*	ABL
53	ICGV 02266	(ICGV 94143 x ICGV 94136) F2-B1-B1-B1-B1-B1	DR	ICRISAT, Hyderabad	Spanish bunch	*fastigiata*	ABL
54	ICGV 13189	{ICGV 91114-P1 x [ICGV 91114-P1 x (ICGV 91114-P1 x GPBD 4-P1_13–1)]} BC2F1P3-P1-B1-B1-B1	DT	ICRISAT, Hyderabad	Spanish bunch	*fastigiata*	ABL
55	ICGV 13207	{TAG 24- P3 x [TAG 24-P3 x (TAG 24-P3 x GPBD 4-P1_27–1)]} BC2F1P2-P2-B2-B1-B1	FDR	ICRISAT, Hyderabad	Spanish bunch	*fastigiata*	ABL
56	ICGV 14421	(ICGV 91114-P1 x GPBD 4-P2-16-7) F2-P13-P29-B2-B2-B1-B1-B1	FDR	ICRISAT, Hyderabad	Spanish bunch	*fastigiata*	ABL
57	ICGV 13219	{JL 24- P1 x [JL 24- P1 x (JL 24-P1 x GPBD 4-P1_19–5)]} BC2F1P1-P6-B1-B2-B1	DT	ICRISAT, Hyderabad	Spanish bunch	*fastigiata*	ABL
58	GPBD 4	KRG 1 x ICGV 86855	FDR	Karnataka, India	Spanish bunch	*fastigiata*	Cultivar
59	ICGV 86031	(F 334 A-B-14 x NC Ac 2214) F2-B1-B3-B2-B3-B2-B3	MD	ICRISAT, Hyderabad	Spanish bunch	*fastigiata*	Cultivar
60	ICGV 16686	ICGV 06110 x (ICGV 06110 x Sun Oleic 95-R) F1P2-BC1F1P3-P9-P4-P28-P2-B1-B1	HOA	ICRISAT, Hyderabad	Spanish bunch	*fastigiata*	ABL
61	ICGV 16005	(ICGV 06420 × Sun Oleic 95R) F2P411-P2-P9-P29-B1-B1	HOA	ICRISAT, Hyderabad	Spanish bunch	*fastigiata*	ABL
62	ICGV 171013	ICGV 07368 x (ICGV 07368 x Sun Oleic 95-R) F1P1-BC1F1P39-P3-P1-P2-P5-P2-B1	HOA	ICRISAT, Hyderabad	Spanish bunch	*fastigiata*	ABL
63	ICGV 171026	(ICGV 00350 x SO 95R)F2 SSD-SSD-SSD-SSD-P18-B1	HOA	ICRISAT, Hyderabad	Spanish bunch	*fastigiata*	ABL
64	ICGV 171039	ICGV 06110x[ICGV 06110x{ICGV 06110x(ICGV 06110 x SO 95R)}]-BC3F1P4-P17-P7-P1-B1-B1-B1	HOA	ICRISAT, Hyderabad	Spanish bunch	*fastigiata*	ABL
65	ICGV 171046	ICGV 06142x[ICGV 06142x{ICGV 06142x(ICGV 06142 x SO 95R)}]-BC3F1P96-P14-P2-P3-B1-B1-B1	HOA	ICRISAT, Hyderabad	Spanish bunch	*fastigiata*	ABL
66	ICGV 181017	((ICGV 06142 x Sun Oleic 95R) x Sunoleic 95-R)-P4-P4-P1-P1-P1	HOA	ICRISAT, Hyderabad	Spanish bunch	*fastigiata*	ABL
67	ICGV 181063	(ICGV 02266 x ICGV 15059)-P2-P1-P1-P1-P1-P1	HOA	ICRISAT,Hyderabad	Spanish bunch	*fastigiata*	ABL
68	ICGV 98412	[(ICGV 88361 x ICGV 88390)x(ICGV 88438 x ICG 5240)F1] F2-SSD-B3-B1-B2-B1-B2-B1-B1	CON	ICRISAT, Hyderabad	Spanish bunch	*fastigiata*	Cultivar
69	ICGV 181489	(ICGV 00351 x Sun Oleic 95R)-14-1-1-1-1-B1	HOA	ICRISAT, Hyderabad	Spanish bunch	*fastigiata*	ABL
70	ICGV 181490	(DH 86 x Sun Oleic 95R)-5-1-1-1-1-B1	HOA	ICRISAT, Hyderabad	Spanish bunch	*fastigiata*	ABL
71	ICGV 92054	[ICGV 87137 x (ICGS 21 x ICGS 50)F5] F2-B1-B1-B1VB-B2SB-B2-B1VB	MD	ICRISAT, Hyderabad	Virginia bunch	*hypogaea*	ABL
72	ICGV 93162	[ICGV 86187 x (JL 24 x Robut 33–1)] F2-B1-B1-B1-B2-B1-B1-B1-B1	MD	ICRISAT, Hyderabad	Virginia bunch	*hypogaea*	ABL
73	ICGV 95111	(ICGV 88308 x ICGMS 42) F2-SSD-SSD-SSD-B2SB-B1-B2-B1	MD	ICRISAT, Hyderabad	Virginia bunch	*hypogaea*	ABL
74	ICGV 96165	(CSMG 84–1 x ICGS 76) F2-SSD-SSD-SSD-B4-B1-B1	MD	ICRISAT, Hyderabad	Virginia bunch	*hypogaea*	ABL
75	ICGV 97115	(ICGV 88308 x CSMG 84–1) F2-SSD-B1-B1-B1-B1-B1	MD	ICRISAT, Hyderabad	Virginia bunch	*hypogaea*	ABL
76	ICGV 98184	(ICGV 91061 x ICGV 86015) F2-SSD-SSD-B1NI-B1-B1-B1	MD	ICRISAT, Hyderabad	Virginia bunch	*hypogaea*	ABL
77	ICGV 01491	[(ICGV 88414 x USA 63) x ICGV 95172] F2-SSD-B2-B1-P1-B1-B2-B1-B1	MD	ICRISAT, Hyderabad	Virginia bunch	*hypogaea*	ABL
78	ICGV 03287	(ICGV 99229 x ICGV 97245) F2-P21-P3-P1-B1	MD	ICRISAT, Hyderabad	Virginia bunch	*hypogaea*	ABL
79	ICGV 05057	{[ICGV 86015 x (B4 x ICGMS 2)] x (ICGV 92035 x ICGV 93128)} F2-SSD-SSD-B1-B1-B1-B1-B1-B1-B1-B2	MD	ICRISAT, Hyderabad	Virginia bunch	*hypogaea*	ABL
80	ICGV 06175	(ICGV 99052 x ICGV 00241) F2-B1-SSD-P1-B1-B1-B1	FDR	ICRISAT, Hyderabad	Virginia bunch	*hypogaea*	ABL
81	ICGV 00064	{[ICGV 88312 x (B4 x ICGV 86885)] x [(JL 24 x ICG(FDRS) 4) x JL 24]} F2-SSD-SSD-SSD-B1-B1-B2(VB)	FDR	ICRISAT, Hyderabad	Virginia bunch	*hypogaea*	ABL
82	ICGV 00246	(ICGV 93222 x ICGV 92209) F2-P6-B1-B1-B2-B1-B1(VB)	FDR	ICRISAT, Hyderabad	Virginia bunch	*hypogaea*	ABL
83	ICGV 97150	{[([(JH 60 x PI 259747)-F2-B1-B1-B2-B2-B1-B1 x NC Ac 17133]F2-B2-B2-B1-B1-B1 x J 11)x NC Ac 343]x ICGV 86003} F2-B1-B1-B1-B1-B1-B1-B1-B1-B1	FDR	ICRISAT, Hyderabad	Virginia bunch	*hypogaea*	ABL
84	ICGV 98385	(91/57-2 x PI 270806) F2-P13-B1-B1-B2-B1-B2-B1	FDR	ICRISAT, Hyderabad	Virginia bunch	*hypogaea*	ABL
85	ICGV 96266	(ICGV 86577 x ICGV 86594) F2-B1-B1-B2-B1-B2-B1-B1-B1-B1-B1-B1-B1	FDR	ICRISAT, Hyderabad	Virginia bunch	*hypogaea*	ABL
86	ICGV 14224	(ICGV 06184 x ICGV 07076) F2-SSD-SSD-P4-B1-B1	FDR	ICRISAT, Hyderabad	Virginia bunch	*hypogaea*	ABL
87	ICGV 14232	[(ICGV 00037 x ICGV 00038) x ICGV 06184] F2-SSD-SSD-P2-B1-B1	FDR	ICRISAT, Hyderabad	Virginia bunch	*hypogaea*	ABL
88	ICGV 07262	[(ICGV 92069 x ICGV 93184)SIL 4 x (ICGS 44 x ICGS 76)] F2-SSD-SSD-P13-B1-B1-B1	DT	ICRISAT, Hyderabad	Virginia bunch	*hypogaea*	ABL
89	ICGV 07247	[(ICGV 92069 x ICGV 93184)SIL 4 x (ICGS 44 x ICGS 76)] F2-SSD-SSD-P12-B1-B1-B1	DT	ICRISAT, Hyderabad	Virginia bunch	*hypogaea*	ABL
90	ICGV 10371	{{[(ICGV 87121 x ICGV 87853) x ICGV 93023] x ICGV 99160}B1 x [ICGV 87846 x (ICGV 87290 x ICGV 87846)]B1VB}} F2-SSD-SSD-P2-B1-B1-B1-B1-B1-B1	DT	ICRISAT, Hyderabad	Virginia bunch	*hypogaea*	ABL
91	ICGV 10373	{{[(ICGV 87121 x ICGV 87853) x ICGV 93023] x ICGV 99160}B1 x [ICGV 87846 x (ICGV 87290 x ICGV 87846)]B1VB}} F2-SSD-SSD-P2-B1-B2-B1-B1-B1-B1	DT	ICRISAT, Hyderabad	Virginia bunch	*hypogaea*	ABL
92	ICGV 10379	(ICGV 03115 x ICGV 91114) F2-SSD-SSD-P7-B1-B1-B1-B1-B1	DT	ICRISAT, Hyderabad	Virginia bunch	*hypogaea*	ABL
93	ICGV 15094	(ICGV 06420 × Sun Oleic 95R)F1P8-BC1F1P28-P3-P6-P25-P10-B1	HOA	ICRISAT, Hyderabad	Virginia bunch	*hypogaea*	ABL
94	ICGV 87846	(CS 9 x ICGS 5) F2-B1-B2-B2-B1	DT	ICRISAT, Hyderabad	Virginia bunch	*hypogaea*	Cultivar
95	ICGV 86699	CS 29/1-B2-B1	FDR		Virginia bunch	*hypogaea*	ABL
96	GG 20	GAUGG 10 x Robust 33–1	MD	Gujarat, India	Virginia bunch	*hypogaea*	Cultivar
97	ICGV 171007	ICGV 06110 x (ICGV 06110 x Sun Oleic 95-R) F1P2-BC1F1P11-P7-P1-P6-P2-P2-B1	HOA	ICRISAT, Hyderabad	Virginia bunch	*hypogaea*	ABL
98	ICGV 171027	(ICGV 03042 X SO 95R)F2 SSD-SSD-SSD-SSD-P8-B1	HOA	ICRISAT, Hyderabad	Virginia bunch	*hypogaea*	ABL
99	ICGV 181006	((ICGV 06420 x Sun Oleic 95R) x Sunoleic 95-R)-P1-P15-P1-P2-P1	HOA	ICRISAT, Hyderabad	Virginia bunch	*hypogaea*	ABL
100	ICGV 181033	((ICGV 03042 x Sun Oleic 95R) x ICGV 03042)-2-1-1-1-1	HOA	ICRISAT, Hyderabad	Virginia bunch	*hypogaea*	ABL

DT = drought tolerant, Con = confectionery, FDR = foliar disease resistant, MD = medium maturity, EM = early maturity, MDR = multiple disease resistant, HO = high oil content, HOA = high oleic acid content (78±2%), ABL = advanced breeding line; ICGV = ICRISAT Groundnut Variety.

### Site description

The 100 genotypes were evaluated during 2018/19 and 2019/20 post-rainy seasons at the International Crops Research Institute for the Semi-Arid Tropics (ICRISAT), Patancheru, India. ICRISAT is situated at a latitude of 17.51^0^ N and a longitude of 78.27^0^ E with an altitude of 545m above sea level. The study used genotypes comprising diverse lines, and advanced breeding lines acquired from ICRISAT. The majority of these genotypes are currently used in the groundnut breeding program in Ethiopia, and the remaining lines were recently developed by ICRISAT and believed to be suitable to Ethiopian agro-ecologies.

### Phenotyping

**Fig 1:**
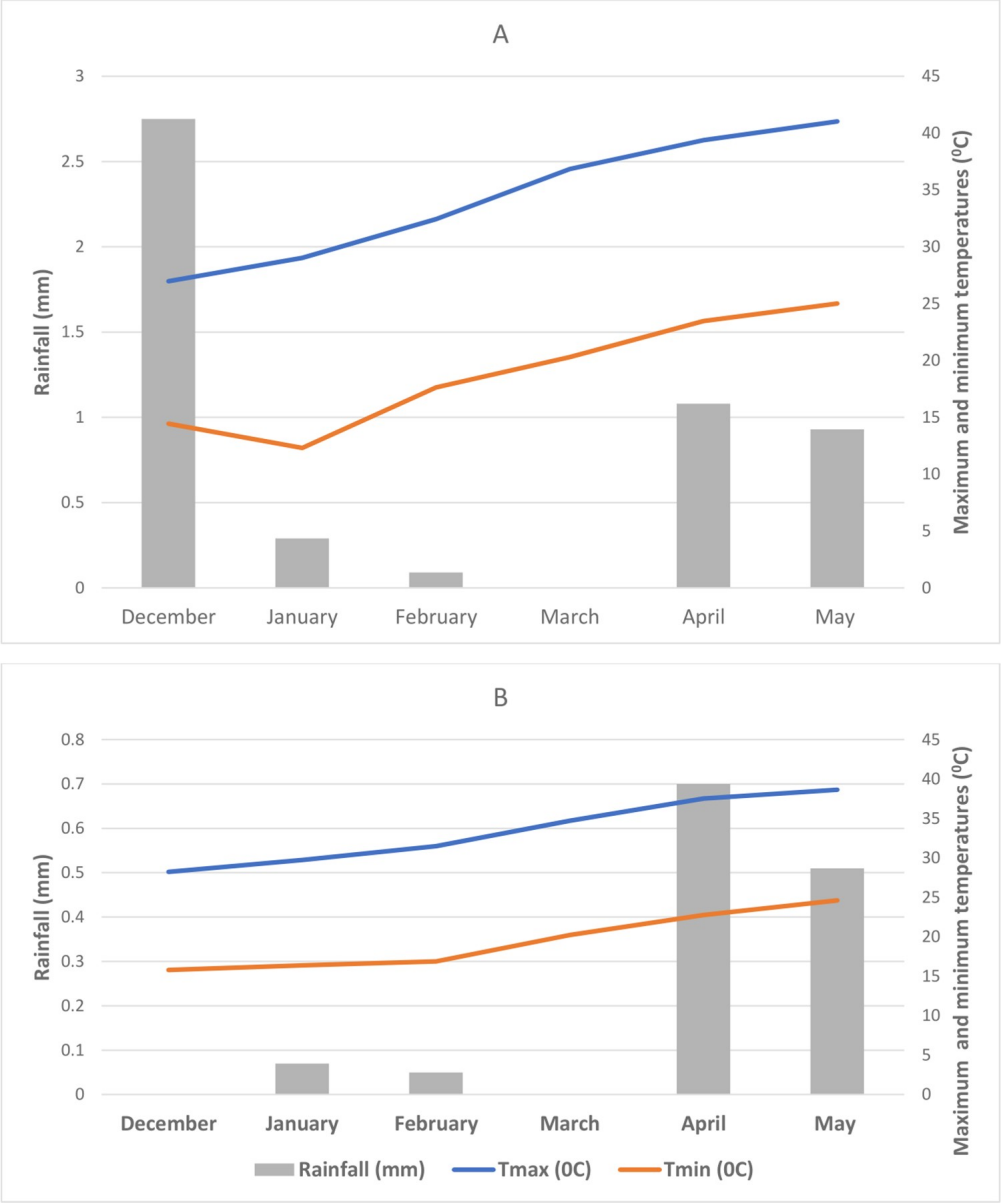
Monthly weather data during the field trial at ICRISAT/India during (A) 2018/19 and (B) 2019/20 post-rainy seasons.

Test genotypes were phenotyped under drought-stressed and non-stressed conditions. The experiments were laid out in a 10 x10 alpha lattice design with two replications. Seeds were sown in 4 rows of 4-meter-long with 30 cm between rows and 10 cm between plants. The non-stressed experiment was maintained with regular irrigation and, drought-stress was imposed from flowering to physiological maturity by with-holding irrigation until wilt symptoms appeared [39]. All the recommended agronomic practices and plant protection measurements were applied [40]. Weather data for the period are presented in Fig 1. During the 2018/19 and 2019/20 post-rainy seasons, the mean minimum and maximum temperatures were 18.86/34.28 and 19.45/33.39 ^0^C.

### Data collected

Data on the following phenotypic traits were collected. Days to 50% flowering (DF) were recorded by counting the number of days from sowing to when 50% of the total plant stand had reached flowering. Soil plant analysis development (SPAD) chlorophyll meter reading (SCMR) was recorded at 80 days after sowing from each plant’s second, third, and fourth trifoliate leaves between 8:00 to 9:30 am. The SCMRs were recoded using a Minolta SCMR-502 m (Tokyo, Japan), and the reading were recorded as described by Nageswara Rao et al. [41]. Leaf area was measured using a leaf area scanner, and leaves were oven-dried at 80 ^0^C for 48 hours. Specific leaf area (SLA) was calculated based on the formula suggested by Nageswara Rao et al. [41] as follow:

$$SLA=Leaf\;area\;\left(cm^{2}\right)/Leaf\;dry\;weight\;\left(g\right)$$

Leaflets of five plants were collected and stored in sealed plastic bags and transported to the laboratory for fresh weight measurement. After the fresh weight measurements, the samples were soaked in distilled water for 8 hours, and accordingly, saturated weights were recorded. Then, leaf relative water content was calculated according to the formula given by Gonzalez and Gonzalez, [42]:

$$LRWC=\frac{fresh\;weight‐dry\;weight}{Saturated\;weight‐dry\;weight}X\;100$$

Plant height (PH, expressed in cm) was measured from ten randomly sampled and tagged plants from the soil surface to the tip of the main stem. The number of primary branches (PB) was recorded as the average number of primary branches from the ten plants. Pod yield per plant (PY, expressed in g plant^-1^) was recorded as the average pod weight of ten sample plants. Pods of each genotype were assorted using a sieve and size ranges between 6 to 8mm were selected. A random sample of pods weighing 200g was used to calculate shelling percentage which was a proportion of shelled seed weight to the unshelled pods. Seed yield per plant (SY, expressed in g plant^-1^) was estimated as the product of pod yield per plant and shelling percentage. Total biomass per plant (TBM, expressed in g plant^-1^) was recorded as the mean total biomass weight of ten sample plants during the physiological maturity of the crop. Harvest index (HI) was computed as a ratio of pod weight to total biomass [43].

### Phenotypic data analysis

Analysis of variance was performed using SAS version 9.3 Software. Genotypes were treated as fixed effects, whereas replications and block nested within replications were fitted as random effects. Pearson correlation was performed using SAS software. Principal component analysis was carried out using JMP Version 15.1 Software with mean observation of all the traits.

### Genotyping

Hundred groundnut genotypes were grown under field conditions at ICRISAT, Hyderabad, India. Genomic DNA was extracted from leaves of three weeks old seedlings at the Center of Excellence in Genomics and Systems Biology at ICRISAT. DNA was extracted using the modified cetyl trimethyl ammonium bromide (CTAB) method [44]. DNA was quantified by loading 1 μl DNA on the 0.8% agarose gel containing 10 ml ethidium bromide (10 mg/ml) and run at 80 V for 30–45 min. The agarose gel was documented under a UV transilluminator. DNA quality and concentration were estimated using NanoDrop Spectrometry (UV 160 A, Japan). Haplotype-based genotyping using 48k SNP Array was conducted at the University of Georgia, Tifton, United States [45].

### Data analysis

SNP data were analyzed using the Axiom analysis suite [46]. SNP markers with more than 20% of missing data and the minor allele frequencies lower than 0.05 were eliminated, resulting in 16,363 SNP markers, which were used for further analysis [47]. Ninety-nine genotypes (except ICGV 06420) were used after the data imputation. Genotype ICGV 06420 was discarded from the analysis due to a low quantity of DNA. The genotype data filtering was performed using TASSEL version 5.2.61 software [48]. Genetic dissimilarity, minor allele frequency (MAF), observed gene diversity, polymorphic information content (PIC), and inbreeding coefficients were determined using GenALEx Version 6.5 Software [49]. The Inbreeding coefficients were determined according to the protocol developed by Nei and Li [49] using GenALEx Version 6.5 Software [50]. Analysis of molecular variance was performed using GenALEx version 6.5 Software to estimate fixation (F_ST_) values and partition molecular variance within sub-species and among subspecies of cultivated groundnut. The genetic differentiation parameter (PhiPT) was used to measure the similarity of pairwise genotypes from the entire collection. Phi’PT represents the proportion of PhiPT relative to the maximum variability proportion attainable PhiPTmax calculated as PhiPT/PhiPTmax [51]. The pairwise Nei genetic distance matrix between 99 genotypes was analyzed using TASSEL Software. The population structure pattern and admixture detection were inferred using a Bayesian model-based clustering algorithm implemented in STRUCTURE version 2.3.4 [52]. The length of the burn-in period and Markov Chain Monte Carlo (MCMC) were set at 10,000 iterations [53]. The K value was set between 1 and 10 to generate the number of subpopulations in the genotypes. Twenty runs were performed for each K-value to obtain an accurate estimation of the number of populations. Delta K values were calculated, and the appropriate K value was determined by the Evanno et al. [53] method using the STRUCTURE Harvester program [54].

### Joint analysis of phenotypic and genotypic data

A joint analysis based on a combination of phenotypic and genotypic dissimilarity matrices was conducted. A phenotypic distance matrix was generated using gower’s distance matrix, while genotypic dissimilarity matrix was generated based on Jaccard’s Coefficient. A joint matrix was performed based on the summation of phenotypic and genotypic matrices. The phenotypic, genotypic, and combined matrix were used to generate hierarchical clustering in the package R software [55]. A Comparison of phenotypic and genotypic hierarchical clusters was assessed using the tanglegram function in the dendextend R package [56].

## Results

### Genetic variation among groundnut genotypes

Analysis of variance for 13 phenotypic traits of 100 groundnut genotypes evaluated under drought-stress and non-stressed conditions are presented in Table 2. Under drought-stressed conditions, the ANOVA revealed significant (p<0.05) difference among genotypes for plant height (cm), SCMR, specific leaf area (cm ^2^ g^-1^) and shelling percentage, and highly significant differences(p<0.001) for days to 50% flowering, number of primary branches, leaf relative water content, haulm weight (g plant^-1^), hundred seed weight (g), pod weight (g plant^-1^), total biomass weight (g plant^-1^) and harvest index (%). Under optimum conditions, the result showed non-significant differences for SCMR and SLA; significant differences for the number of primary branches, and highly significant differences for the rest of the tested traits. A non-significant difference for genotype by year interaction was recorded for SCMR and SLA under both moisture stress conditions. The highest pod yield per plant under drought-stressed condition was recorded for ICGV 01260 (8.57g), ICGV 06040 (8g), ICGV 06175 (7.51g), ICGV 07222 (7.2g), and ICGV 10178 (7.12g) while ICGV 98412 (16.21g), ICGV 07222 (15.93g) and ICGV 10143 (15.49g) were under non-stressed conditions (S1 and S2 Tables).

**Table 2:** Analysis of variance showing mean square values due to year, replications (Rep), blocks (BLK), genotypes (Geno), and genotype by year and error 13 phenotypic traits among 100 groundnut genotypes across two seasons evaluated under drought-stressed and non-stressed conditions.

Drought-stressed							Non-stressed					
traits	Year	rep(year)	Blk(year*rep)	Geno	year*Geno	Error	Year	Rep(year)	Blk(year*rep)	Geno	Year*Geno	Error
**DF**	10826.40**	9.03*	1.17ns	3.48**	2.20**	1.01	14137**	20.29**	2.60*	3.37**	2.45*	1.56
**PH**	11509**	9.95^NS^	7.63*	7.13*	5.49^NS^	4.27	21744**	192.66**	6.31^NS^	26.07**	12.77*	7.92
**PB**	42.45**	9.52*	3.28*	4.77**	1.7^NS^	1.68	244**	10.23^NS^	2.39^NS^	7.84*	4.12^NS^	4.46
**SCMR**	2218.02**	112.58^NS^	109.22^NS^	124.42*	106.43^NS^	87.57	2.13^NS^	120.27*	19.42^NS^	35.55^NS^	15.45^NS^	14.62
**LRWC**	11391**	45.39^NS^	209.80ns	146.58**	125.66^NS^	214.4	11392**	709.52**	98.31^NS^	239.03**	224.79**	67.15
**SLA**	1647.28*	200.18^NS^	209.98ns	206.51*	173.13^NS^	164.2	1647*	1145*	114^NS^	228^NS^	236^NS^	241.29
**HUALM**	117.84*	165.59**	30.06**	38.08**	21.23*	11.48	6275**	566**	32.51^NS^	57.55**	32.36^NS^	26.89
**SHP**	4043.68**	146.34^NS^	36.58ns	45.28*	40.52^NS^	48.33	4044**	9.20^NS^	11.64^NS^	103.31**	100.26**	11.25
**HSW**	1228**	23.18^NS^	16.89ns	26.23**	24.18^NS^	19.3	1228**	96.59**	10.08^NS^	52.68**	52.32**	8.92
**PY**	3586**	29.57**	1.50^NS^	6.48**	4.46**	1.65	1114**	107**	11.97^NS^	21.78**	14.32*	8.27
**TBM**	5950**	161.52**	32.85*	98.17**	76.14**	13.64	2102**	1043**	65.72^NS^	65.37*	58.39ns	49.35
**HI**	38776**	340.56**	19.22^NS^	89.52**	41.60**	16.33	17532**	165.81*	33.31^NS^	155.52**	56.41**	28.08
**SY**	1120.52**	4.00*	0.75^ns^	2.23**	1.48**	0.68	126.93**	33.33**	4.55 ^NS^	9.1**	6.05**	3.07

d.f. = degree of freedom, rep = replications, blk = no of blocks, trt = number of treatment, DF = days to 50% flowering, PH = plant height (cm), PB = number of primary branches, SCMR = SPAD chlorophyll meter reading, HAULM = haulm weight (g/plant), SHP = shelling percentage, SLA = specific leaf area (cm^2^/g), LRWC = leaf relative water content, HSW = hundred seed weight (g), PY = pod yield (g/plant), TBM = Total biomass (g/plant), HI = harvest index (%), SY = seed yield (g/ plant, NS = non-significant

*, ** significant level at 5% and 1% probability level, respectively.

### Association of traits

Pearson correlation among the studied traits is summarized in Table 3. The correlation result revealed that harvest index and total biomass per plant were positively and significantly associated with pod yield per plant under both drought-stressed and non-stressed condition. Under drought-stressed condition, PY showed significant (p ≤ 0.05) correlation with SY (r = 0.97), HI (r = 0.92), TBM (r = 0.55) and SHP (0.38), HSW (r = 0.36), LRWC (r = 0.26) and SLA (r = 0.13), while under non-stressed condition, PY exhibited significant (p ≤ 0.05) correlation with SY (r = 0.93), HI (r = 0.81) and TBM (r = 0.35). The following traits revealed significant (p ≤ 0.05) correlations: SHP and HSW (r = 0.48), PH and SHP (0.36), PLRWC and HSW (r = 0.47) under drought-stressed and DF and PB (r = 0.45), DF and HLM (r = 0.24) under optimum condition.

**Table 3:** Pearson’ s correlation coefficient (r) showing association of 13 phenotypic and physiological traits of 100 groundnut genotypes evaluated across two seasons evaluated under drought-stresses (upper diagonal) and non-stressed (lower diagonal) conditions.

	DF	PH	PB	SCMR	HAULM	PY	TBM	HI	SHP	HSW	LRWC	SLA	SY
DF	1	-0.88**	-0.12*	0.21**	-0.01ns	-0.81**	-0.41**	-0.81**	-0.43**	-0.35**	-0.36**	-0.12*	-0.11ns
PH	0.09ns	1	0.17*	-0.24**	0.12*	0.78**	0.43**	0.77**	0.36**	0.31**	0.32**	0.19*	0.0002ns
PB	0.47**	0.31*	1	-0.03ns	0.15*	0.15*	0.19*	0.11*	-0.01ns	0.04ns	0.06ns	-0.07ns	-0.19ns
SCMR	0.09ns	-0.09ns	-0.16ns	1	-0.001ns	-0.2**	-0.08ns	-0.21**	-0.07ns	-0.027ns	-0.07ns	-0.041ns	-0.12ns
HAULM	0.24*	0.38**	0.39**	-0.06ns	1	0.14*	0.67**	-0.17*	0.02ns	-0.005ns	0.01ns	0.1ns	-0.009
PY	-0.2*	-0.08ns	-0.08ns	0.05ns	-0.22*	1	0.55**	0.92**	0.38**	0.36**	0.28**	0.13*	0.97**
TBM	0.12ns	0.32*	0.34*	-0.03ns	0.84**	0.35*	1	0.25**	0.22**	0.12*	0.21**	0.10*	0.34*
HI	-0.31*	-0.29*	-0.31*	0.01ns	-0.72**	0.81**	-0.24*	1	0.37**	0.37**	0.29**	0.12*	0.78**
SHP	0.17ns	-0.11ns	0.01ns	0.09ns	-0.3*	0.18ns	-0.19ns	0.25*	1	0.48**	0.48**	0.17*	0.54**
HSW	0.14ns	-0.1ns	0.10ns	-0.09ns	-0.06ns	0.17ns	0.04ns	0.17ns	-0.07ns	1	0.47**	0.36**	0.3*
LRWC	-0.03ns	-0.0005ns	-0.01ns	-0.09ns	-0.05ns	-0.05ns	-0.07ns	0.008ns	-0.05ns	0.36*	1	0.19*	-0.03ns
SLA	0.001ns	-0.03ns	-0.1ns	-0.10ns	-0.07ns	0.025ns	-0.05ns	0.1ns	0.14ns	0.05ns	-0.07ns	1	-0.05ns
SY	-0.11ns	-0.09ns	-0.05ns	0.06ns	-0.3*	0.93**	0.24*	0.79**	0.52**	0.21*	0.3*	0.09ns	1

DF = days to 50% flowering, PH = plant height (cm), PB = number of primary branches, SCMR = SPAD chlorophyll meter reading, HUALM = haulm weight (g/plant), PY = pod yield (g/plant), TBM = Total biomass (g/plant), HI = harvest index (%), SHP = shelling percentage, SLA = specific leaf area (cm^2^/g), LRWC = leaf relative water content HSW = hundred seed weight (g), SY = seed yield per plant, NS = non-significant

*, ** significant level at 5% and 1% probability level, respectively.

### Principal component (PC)

The first five PCs with Eigenvalues greater than one accounted for 75.59% and 77.70% of the total phenotypic variability exhibited by the studied traits under drought-stressed and optimum conditions, respectively (Table 4). DF, PH, and HI were the main contributing traits in PC1 under both moisture conditions, and HLM and TBM in PC2 under drought stress conditions, and PY and TBM under non-stressed conditions. PY was one of the main contributing traits in PC1 under drought-stressed conditions and in PC2 under non-stressed conditions.

**Table 4:** Principal component scores, Eigenvalues, variances of 13 phenotypic traits among 100 groundnut genotypes evaluated under drought-stressed and non-stressed conditions across two seasons.

Drought-stressed						Non-stressed				
Traits	PC1	PC2	PC3	PC4	PC5	PC1	PC2	PC3	PC4	PC5
DF	-0.40	0.41	0.04	0.53	-0.24	-0.30	0.49	-0.34	0.14	-0.42
PH	-0.11	0.18	-0.06	-0.50	-0.22	-0.40	0.36	0.18	-0.13	0.07
PB	-0.44	0.31	0.02	0.49	0.06	-0.40	0.60	-0.03	0.05	-0.42
SCMR	-0.24	0.06	-0.24	0.11	0.83	0.12	-0.11	0.02	0.80	0.46
LRWC	0.10	-0.13	0.68	0.38	0.04	0.31	0.53	-0.48	-0.01	0.13
SLA	-0.12	0.23	0.57	-0.58	0.08	0.20	0.18	-0.30	-0.59	0.52
HAULM	-0.49	0.81	0.07	-0.06	-0.03	-0.75	0.45	0.30	0.02	0.26
PY	0.78	0.56	-0.19	-0.03	0.09	0.70	0.32	0.62	-0.02	-0.05
SHP	0.62	0.06	0.24	0.27	-0.31	0.54	0.46	-0.48	0.21	0.00
SY	0.84	0.52	-0.12	0.04	0.01	0.81	0.45	0.36	0.05	-0.05
HSW	0.42	0.08	0.69	0.00	0.34	0.24	0.50	-0.45	0.03	0.18
TBM	-0.17	0.94	0.00	-0.07	0.01	-0.33	0.62	0.64	0.01	0.22
HI	0.91	-0.01	-0.23	0.08	0.08	0.91	-0.07	0.27	-0.10	-0.18
Eigenvalue	3.42	2.52	1.50	1.36	1.03	3.57	2.41	1.99	1.09	1.04
Proportion variance (%)	26.34	19.36	11.53	10.44	7.92	27.45	18.51	15.33	8.38	8.03
Cumulative variance (%)	26.34	45.70	57.23	67.67	75.59	27.45	45.96	61.29	69.67	77.70

DF = days to 50% flowering, PH = plant height, PB = number of primary branches per plant, SCMR = SPAD chlorophyll meter reading, LRWC = leaf relative water content, SLA = specific leaf area (cm^2^/g), HAULM = haulm weight per plant, SHP = shelling percentage, HSW = hundred seed weight(g), PY = pod yield per plant, HI = harvest index (%), TBM = total biomass per plant (g), SY = seed yield per plant (g), PC = principal component.

### Genetic variability of 99 groundnut genotypes using SNP markers

Table 5 summarized the diversity indices of 99 groundnut genotypes. The genetic dissimilarity (diversity) (GD) ranged from 0 to 0.5, with a mean of 0.1. The polymorphic information content (PIC) value varied from 0 to 0.38, with a mean of 0.08 per locus. The minor allele frequency ranged from 0 to 0.5, with a mean of 0.08. The lowest and highest observed gene diversity recorded were 0.02 and 0.11, respectively. The inbreeding coefficient (F) ranged from -0.09 to 0.77, with a mean of 0.39.

**Table 5:** Diversity indices statistics of the 99 groundnut genotypes based on 16 363 SNP markers.

Statistics	Genetic parameters				
	GD	PIC	MAF	Ho	F
Minimum	0	0	0	0.02	-0.09
Maximum	0.5	0.38	0.5	0.11	0.77
Mean	0.1	0.08	0.08	0.06	0.39

GD = genetic dissimilarity, PIC = polymorphic information content, MAF = minor allele frequency, Ho = observed gene diversity, F = inbreeding coefficient.

### Genetic relationship among the 99 groundnut genotypes

**Fig 2:**
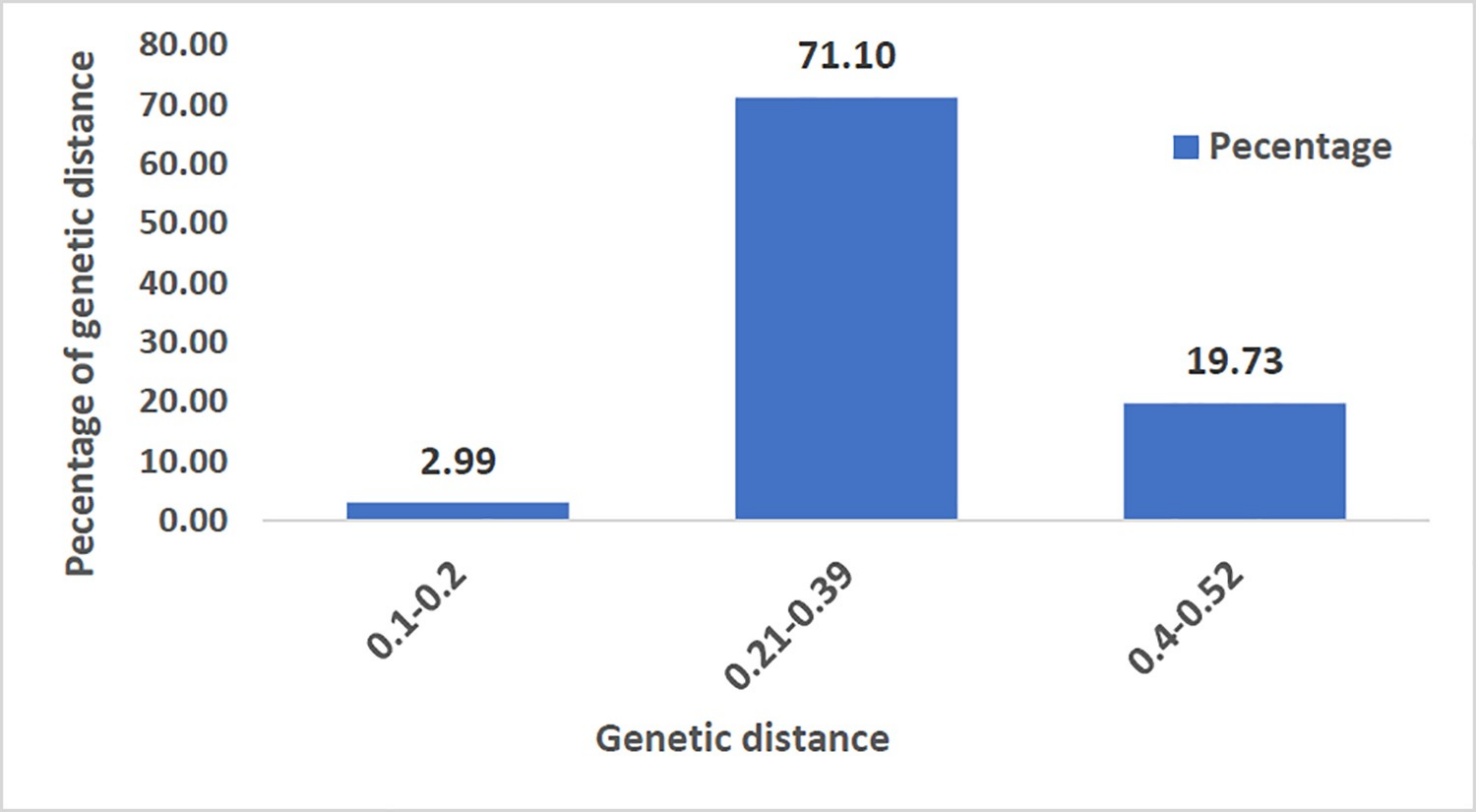
Percentage distribution of pairwise genetic distance for 99 groundnut genotypes with 16,363 SNPs.

The pairwise genetic distance matrix showed 4 851 combinations among the 99 genotypes (S4 Table). The genetic distance ranged from 0.11 to 0.52, with a grand mean of 0.34. Twenty percent of the test genotypes had GD ranging between 0.4 to 0.52, while 71% had a GD ranging from 0.21 to 0.39 (Fig 2). The genetic distance between the two subspecies, *vulgaris*, and *hypogaea*, was similar. The lowest genetic distance (0.11) was observed between ICGV 10371 and ICGV 10373. These two genotypes are categorized under Virginia (var. *vulgaris* subspecies *hypogaea*), and they have good resistance to late leaf spot and rust. The pedigree of these two genotypes revealed common parentage involving ICGV 87846, and with similar selection history. The highest genetic distance (0.52) was observed between ICGV 95111 and ICGV 13189. These genotypes were derived from different genetic backgrounds. ICGV 95111 is a medium maturing genotype, belongs to the Virginia bunch market class, and was derived from a cross between ICGV 88308 x ICGSMS 42. In contrast, ICGV 13189 is a drought-tolerant genotype that belongs to the Spanish (var. *fastigiata* subspecies *vulgaris*) market class and was derived from a cross between ICGV 91114 x GPBD 4.

### Population structure, and kinship analyses

**Fig 3:**
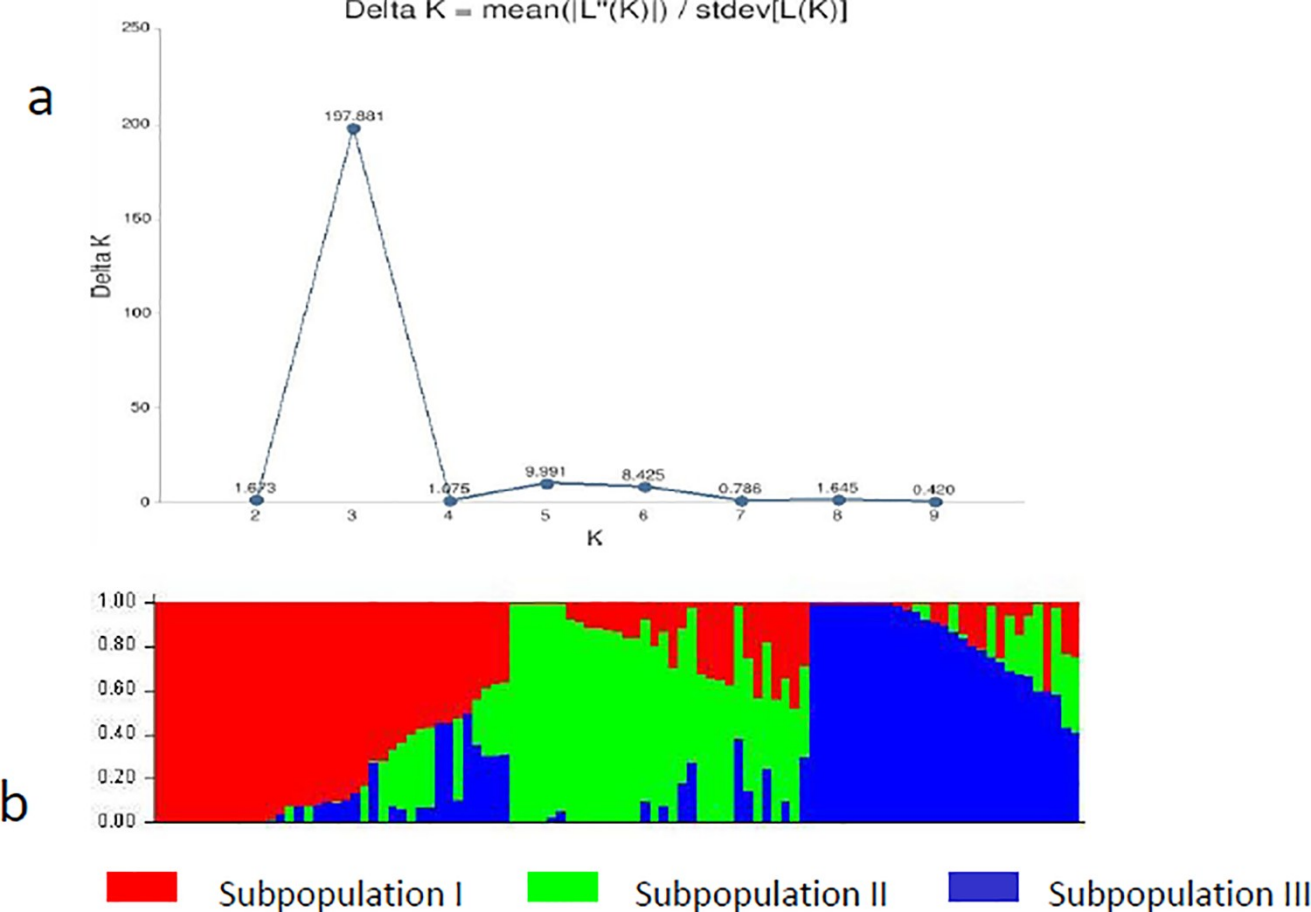
Population structure analysis of 99 groundnut genotypes; (a) Delta K showing the number of populations; (b) Bar plot of population sorted by kinship matrix.

Based on the Evanno method, the best K was estimated to be 3 (Fig 3A), showing three subpopulations (Fig 3B). The population structure analysis revealed three subpopulations with 32% (32/99) admixture genotypes (S5 Table). Allocation into clusters was done at 70% ancestry. Sub-populations 1, 2, and 3 comprised 24, 22, and 21 genotypes, respectively. Subpopulation 1 included 83% Spanish bunch, subpopulation II had 36% Virginia bunch, and subpopulation III consisted of 81% Spanish bunch. Table 6 summarized the allele frequency divergence among subpopulations and expected heterozygosity between the genotypes within the same subpopulations. The highest allele frequency divergence (0.0566) was recorded between subpopulations 1 and 3, followed by subpopulations 2 and 3 with 0.052, while the lowest allele frequency divergence (0.0508) was recorded between subpopulations 1 and 2. The expected heterozygosity among genotypes within the three subpopulations ranged between 0.01 (subpopulation 3) and 0.08 (subpopulation 2), with an average of 0.047.

**Table 6:** Allele frequency divergence among sub-populations and expected heterozygosity (average distance) between genotypes with in the same subpopulations.

	Allele frequency divergence among subpopulations		
	I	II	III
I		0.0508	0.0566
II			0.052
	Expected heterozygosity within subpopulations		
	I	II	III
	0.052	0.08	0.01

I = subpopulation 1, II = subpopulation2 and III = subpopulation 3.

### Genetic differentiation

The analysis of molecular variance (AMOVA) among 99 groundnut genotypes revealed significant differences between the subspecies and within individuals. Nighty-eight percent of the total genetic variation was attributed to differences among individuals, while only 2% of the total variance was due to variation between the subspecies (Table 7). The overall mean PhiPT value was 0.016 (with a maximum value of 0.887 and minimum Phi’PT of 0.018) and an associated permutation P-value <0.05.

**Table 7:** Analysis of molecular variance based on two subspecies using 16,363 SNP markers in 99 groundnut genotypes.

Source	df	SS	MS	Est. Var.	%
Among sub-species	1	1547.995	1547.995	14.799	2%
Within sub-species	97	90123.419	929.107	929.107	98%
Total	98	91671.414		943.907	100%

Note: df = degree of freedom, SS = sum of square, MS = mean square, Est. Var. = estimated variance.

### Combined analysis of phenotypic and genotypic data

**Fig 4:**
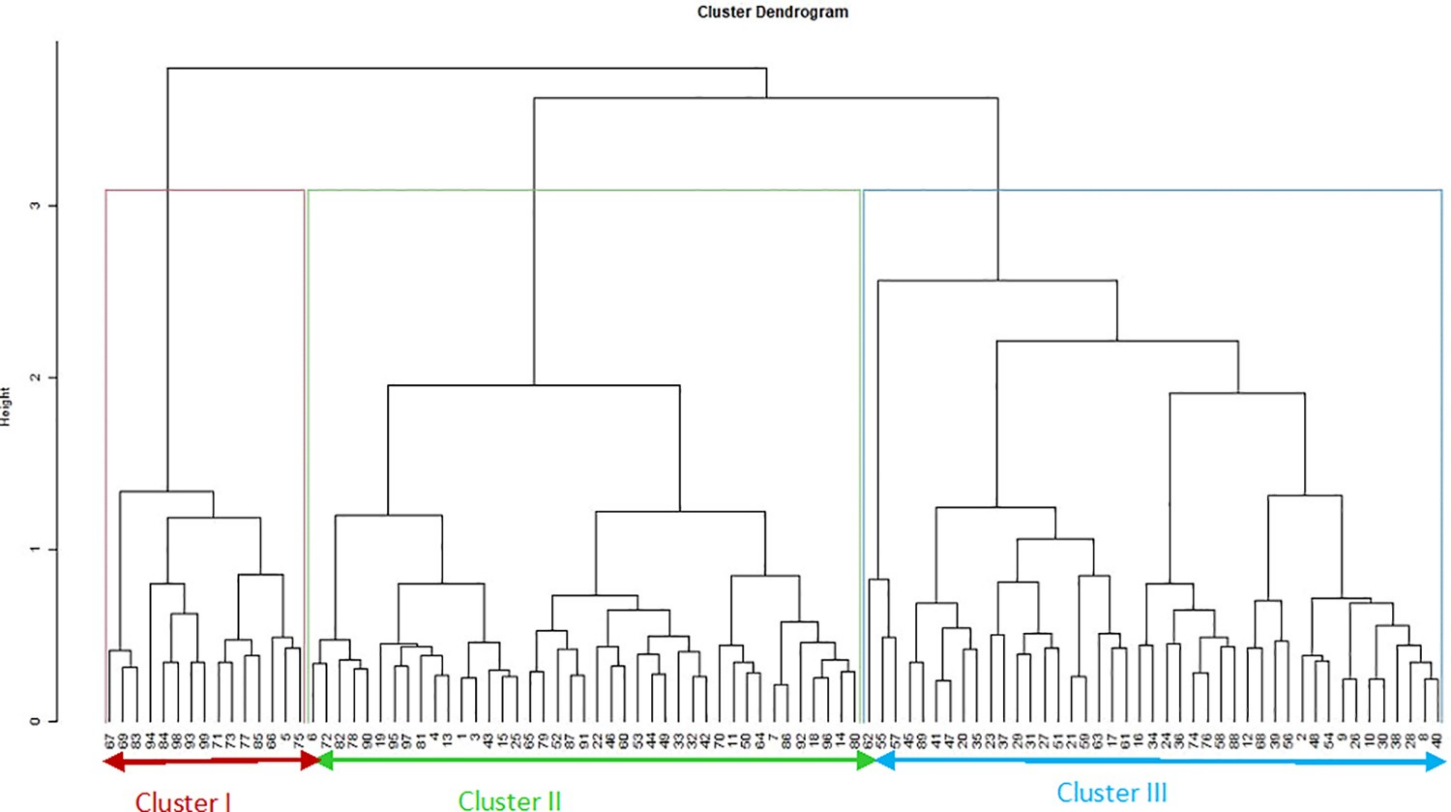
Dendrogram showing relatedness among the 99 groundnut genotypes based on phenotypic matrix.

**Fig 5:**
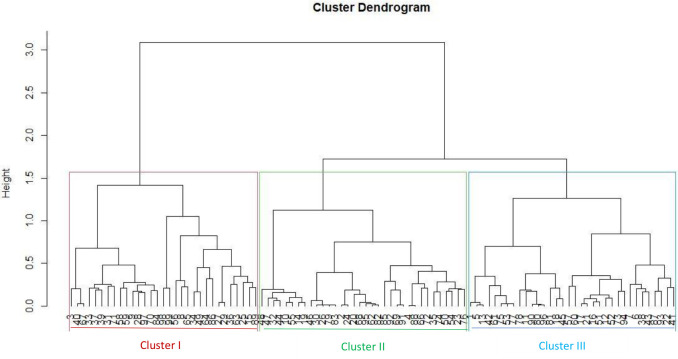
Dendrogram showing relatedness among the 99 groundnut genotypes based on genotypic matrix.

**Fig 6:**
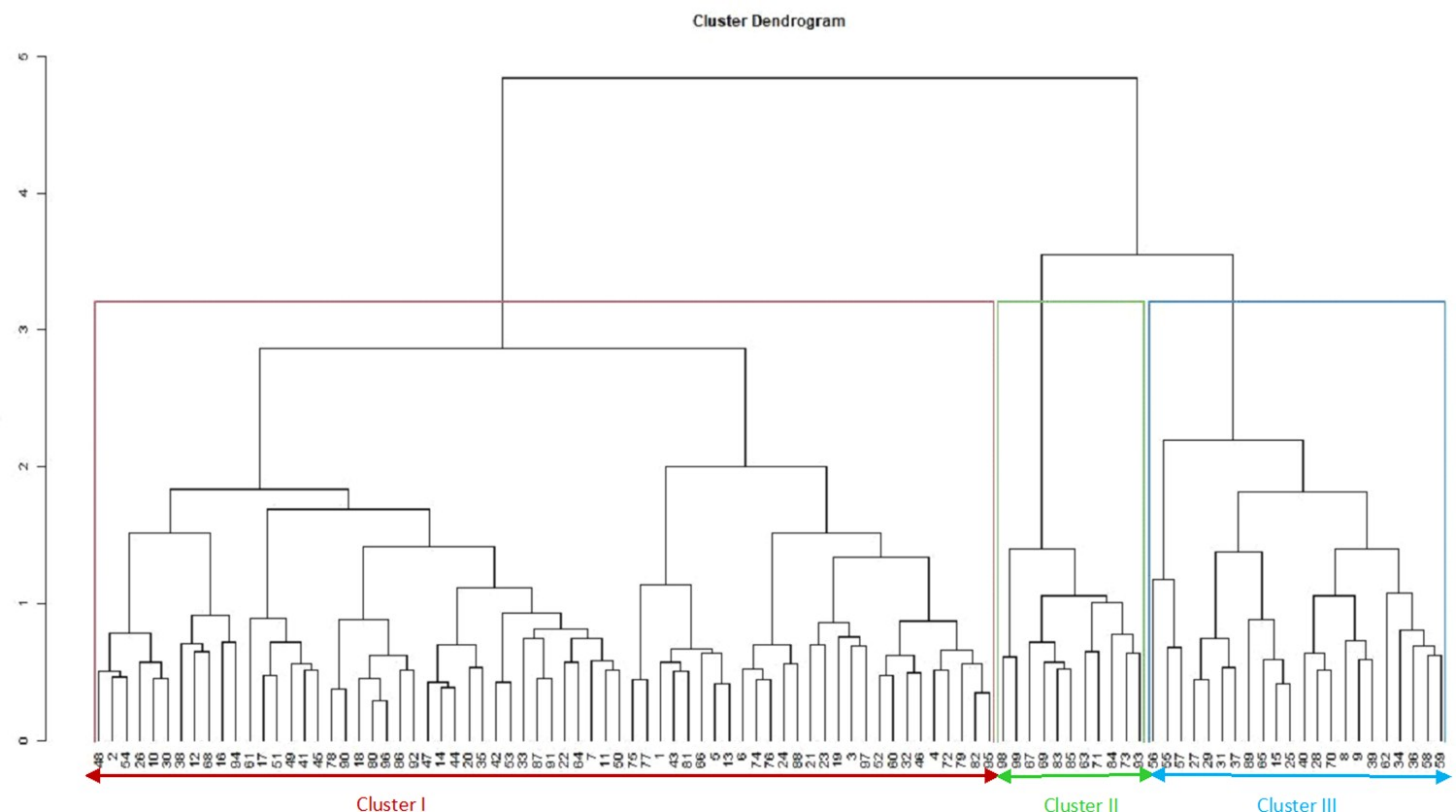
Dendrogram showing relatedness among the 99 groundnut genotypes based on combined matrix.

**Fig 7:**
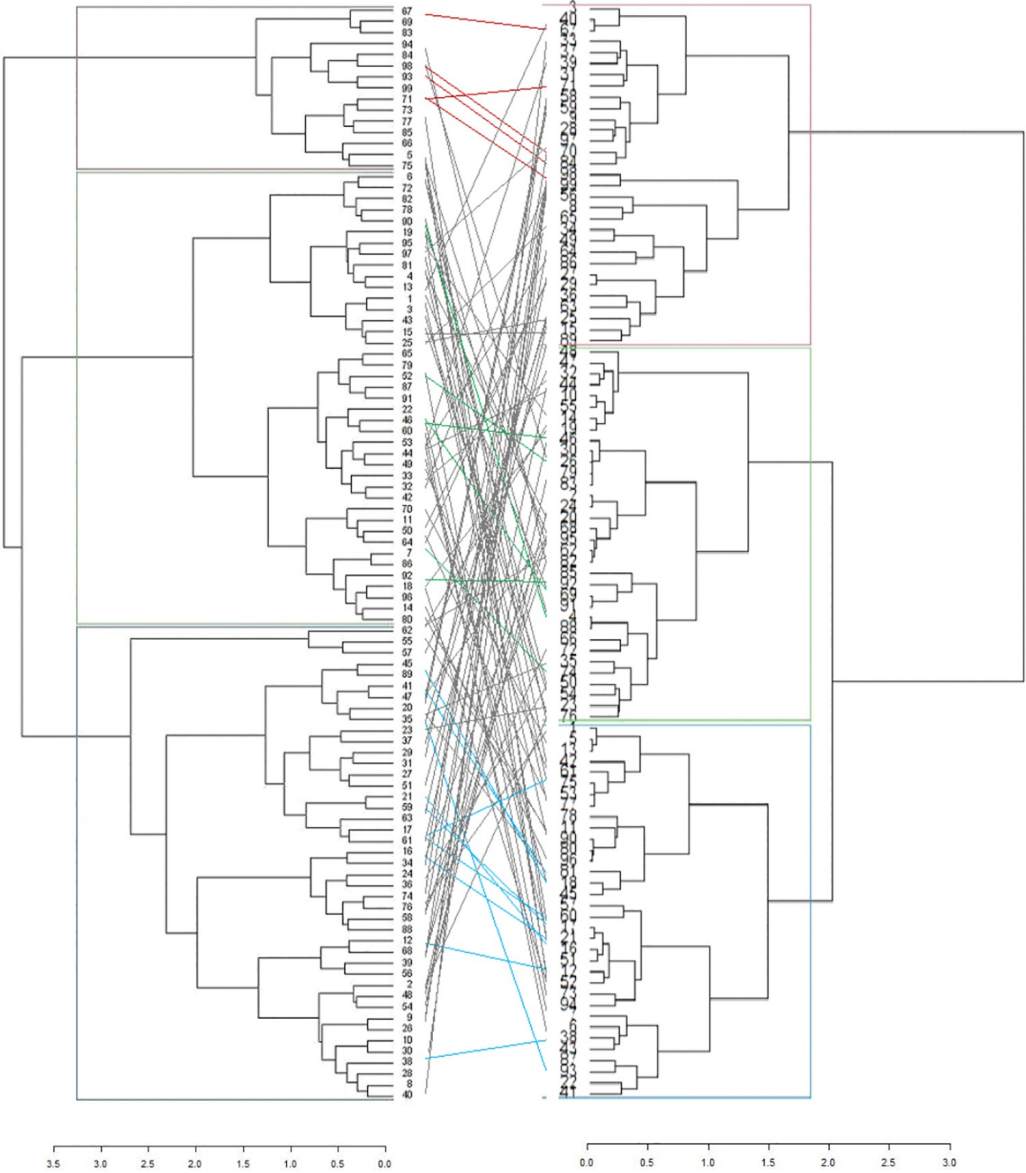
Tanglegram showing comparison of phenotypic and genotypic dendrograms. See codes of genotypes in S5 Table.

The dendrogram based on phenotypic and genotypic data revealed three distinct clusters (Figs 4 and 5). The dendrogram based on phenotypic data showed three clusters: I, II, and III consisting of 40, 63, 93% of the Spanish bunch groundnut types. In cluster I, genotypes ICGV 03042 and ICGV 05155 were included, which have distinguished high oil content. These genotypes are half-sib families with a common ancestor, ICGV 99160. The following full-sib lines: ICGV 0629, ICGV 07262; ICGV 15094, and ICGV 181006 were found in Cluster I. The genotypes ICGV 00187 and ICGV 94118 were allocated in cluster II, with a common ancestor, ICGV 86015. These genotypes are resistant to foliar diseases such as late leaf spot and rust. Cluster III consisted of full-sib lines ICGV 16005, ICGV 181026, and ICGV 15074 with high oleic acid content. The dendrogram based on genotypic data showed that Clusters I, II, and III consisted of 74, 69, and 67% Spanish bunch types, respectively. Using the combined phenotypic and molecular marker data, genetic diversity assessment showed that the test genotypes were allocated into three heterogeneous groups (Fig 6 and S6 and S7 Tables). The tanglegram analysis based on phenotypic and genotypic data set indicated that 21 of the test genotypes maintained their position in both hierarchical clusters (Fig 7).

## Discussion

### Genotypic variation and performance of test genotypes for phenotypic traits

This study evaluated the genetic diversity presented among 100 diverse genotypes of groundnut using phenotypic traits and SNP markers as a preliminary step to identify suitable parental lines for drought tolerance breeding. Analysis of variance revealed significant differences among the genotypes for all the measured traits under drought-stressed and non-stressed conditions except SCMR and SLA under non-stressed conditions, indicating genetic variability for most of the traits among the tested genotypes. Similar findings were reported by Zongo et al. [57], Zaman et al. [58], and Ratnakumar and Vadez [59]. PY, SY, and HI traits were affected by genotype x season interaction under both moisture conditions. In this study, drought stress reduced PY, SY, HI by 44.4%, 40% and 63%, respectively. Pereira et al. [19] reported 32%, 41% and 31% losses in that order.

The knowledge of existing variability and degree of association between yield contributing characters and their relative contribution in yield is essential for developing high yielding genotypes in groundnut [58]. The study selected genotypes ICGV 07222, ICGV 10143, ICGV 06040, ICGV 03042, and ICGV 06175 with high PY under drought-stressed and non-stressed conditions. This suggests that the genotypes can be used in groundnut breeding to exploit their drought tolerance and yield potentials.

### Association studies

The positive and strong association between SY, HI, TBM, HSW, and SHP with pod yield revealed the importance of these characters in determining yield under a drought-stress environment. DF showed a negative and strong correlation with PY and other economic traits such as HI and HSW under drought-stressed conditions, suggesting early flowering provides a promising strategy for developing drought-adapted groundnut cultivars. Similar finds were reported by Zongo et al. [57]. The results identified PY, HI, and SHP as the main contributors to the total variation in SY under both moisture conditions, suggesting these traits could be considered for developing high yielding groundnut cultivars under drought stress and optimum condition. PCA is used to identify traits that contribute to the total variation in a population under a given environment. PY, SY, HSW, SHP, and HI were clustered together in PC 1 and contributed maximum variability for yield under the two water regimes. Hence, selecting these traits will be successful for screening groundnut genotypes under drought-stressed and optimum conditions.

### Genetic diversity estimates based on the SNP markers

Genetic diversity and genetic relationships help minimize the risk of closely related parents, leading to genetic ‘bottlenecks’ in breeding programs [59]. The current study utilized 16,363 SNP markers to elucidate the genetic diversity of 99 groundnut genotypes (S3 Table). Genetic dissimilarity was adopted to measure the genetic divergence among genotypes [60]. In this study, genetic dissimilarity ranged from 0 to 0.5, with an average of 0.1. Similarly, low genetic diversity (0.11) was reported by Ren et al. [34]. Moretzsohn et al. [61] noted that cultivated groundnut presents a relatively low genetic variation when using Random Amplified Polymorphic DNAs (RAPDs), Amplified Fragment Length Polymorphisms (AFLPs), and Restriction Fragment Length Polymorphisms (RFLPs) marker systems. The polymorphism information content (PIC) value is used to measure a genetic marker‘s usefulness for linkage analysis [62]. In this study, PIC value varied from 0 to 0.38, with an average of 0.08. When using SSR markers, this value was relatively lower than a previously reported PIC value of 0.70 [32]. This may be attributed to fewer accessions used in the present study (99) than earlier study (189 accessions) or the differences in the marker types used.

The inbreeding coefficient (F) measures the probability that two alleles at any locus within an individual are identical by descent from the common ancestor(s) of the two parents [63]. If the F value is zero (i.e. as in a random mating system), the genotype frequencies are expected to be at Hardy–Weinberg equilibrium. On the other hand, if the F value is 1, this indicates complete inbreeding with the frequency of heterozygotes being zero [23]. The negative F value indicates the presence of excess heterozygotes. This may be due to high outcrossing or mutation event at a specific locus. In this study, the F value ranged from -0.09 to 0.77, with an average of 0.39, a moderate value for groundnut, a self-pollinating crop. Otyama et al [63] reported negative inbreeding coefficients in groundnut.

A pairwise genetic distance is used to measure genetic variation in a population [64]. The genetic distance estimates ranged from 0.4 to 0.52 for the 25% of test genotypes and 0.1 to 0.2 for 3%. The former genetic distance range indicated that the genotypes under this category are relatively distant or with limited common parentage. The genetic distance between var. *vulgaris* and var. *fastigiata* ranged from 0.11 to 0.52, showing a wide population differentiation between the two sub-species. In contrast, low genetic distances of 0.073 and 0.083 were reported for the two subspecies, in that order [2]. Ren et al. [34] reported the highest genetic distance (0.4) between groundnut genotypes. This result agrees with the current findings. The lowest genetic distance among the cultivars was recorded between ICGV 93260 (Vijetha) and ICGV 93261 (Ajeya). The highest genetic distance (0.4) was observed between Vijetha and GPBD 4. This could be attributed to genetic differentiation involving natural or artificial selection and events such as mutation, genetic drift and gene flow [65]. The most genetically distant genotypes identified in the present study should be used as potential parents in the groundnut breeding program to enhance the genetic base of the available genetic resources and hasten groundnut improvement. In general, the results indicated the availability of considerable genetic diversity among the tested genotypes in the present study.

The genetic population structure reveals genetically distinct subgroups that result from shared ancestry within a large population [66]. The population structure analysis showed three subpopulations, and most genotypes (68%) had a high membership coefficient to their respective subpopulations. This correlates with the findings reported by Daudi et al. [67]. Genotypes with similar genetic backgrounds tended to cluster in the same sub-group, indicating the effectiveness of SNP markers used in this study in assigning the tested genotypes into homogenous groups [37]. Allele frequency divergence measures the magnitude of differentiation between sub-populations. The highest allele frequency divergence was recorded between sub-populations 1 and 3. In contrast, the lowest was recorded between sub-populations 1 and 2, indicating sub-populations 1 and 3 being more divergent than sub-populations 1 and 2. The lower levels of heterozygosity among the tested genotypes within the three sub-populations indicate that the SNP markers effectively constructed homogenous subpopulations [37]. The expected heterozygosity values indicated that sub-population 2 (0.08) had the highest genetic diversity, followed by sub-population 1 (0.05) and sub-population 3 (0.01). Low allele frequency divergence between the two subpopulations could be attributable to possible intercrosses between the two subspecies. Zheng et al. [2] reported nucleotide diversity or expected heterozygosity among three sub-populations with values of 0.048 (population 1 = C1), 0.035 (C2), and 0.012 (C3), values lower than found in the current study.

The AMOVA was done based on the two classes, Spanish and Virginia. Much of the observed differences (98%) were derived from individual differences rather than between the species. This is because the two subspecies have reduced evolutionary gene flow, and only a few genes that regulate growth habits and seed color are the distinguishing features between the two types.

#### Clustering

The molecular genetic diversity study included 99 genotypes, of which 30 and 69 were Virginia bunch and Spanish bunch types, respectively. The combined matrices showed that the groundnut genotypes were clustered into three distinct groups. Most of the Spanish bunch groundnut types were grouped in clusters I and II at a proportion of 70% and 91% in that order, whereas most of the Virginia bunch types (81%) were grouped in Cluster II. Further, Cluster I consisted of drought-tolerant genotypes such as ICGV 00350, ICGV 00351, ICGV 99241, and ICGV 181489. The first three genotypes are full-sibs and were derived from a cross between ICGV 87290 and 87846, while the last genotype, ICGV 181489, has a common ancestor ICGV 00351. ICGV 00351 (CO 7) is a high-yielding variety developed at ICRISAT for cultivation in drought-prone areas [15]. The result from cluster analysis showed partial grouping of accessions based on the two botanical types in agreement with previous findings by Varshney et al. [32] and Otyama et al. [63]. Genotype comparison using the tanglegram showed that 21 groundnut genotypes maintain their position in both phenotypic and genotypic hierarchical clusters. These genotypes are drought-tolerant, resistant to foliar diseases, with high oil and oleic acid contents (Tables 1 and S5). They can be used as desirable parents to broaden the genetic base for multiple traits of interest in groundnut breeding programs.

## Conclusions

This study revealed considerable genetic variation in yield and yield-related components among the tested genotypes evaluated under drought-stressed and optimum conditions. Correlation analyses involving PY, HI, HSW and SHP revealed positive and strong associations with SY under the two water regimes. This provides an opportunity for direct selection to improve yield and drought tolerance in the test genotypes. The negative and strong association between DF and yield and; yield-related components under drought-stress indicates early flowering has an advantage of drought escape during the critical growth stage. The study selected genotypes ICGV 07222, ICGV 06040, ICGV 01260, ICGV 15083, ICGV 10143, ICGV 03042, ICGV 06039, ICGV 14001, ICGV 11380, and ICGV 13200 with high PY under drought-stressed and non-stressed conditions. This aids selecting divergent parental lines for enhanced pod yield.

Clustering based on the Bayesian method grouped the genotypes into three sub-populations. The dendrogram based phenotypic and genotypic data grouped the studied 99 genotypes into three heterogeneous clusters. The information generated in this study provides a detailed understanding of the genetic relationships among the tested genotypes. High genetic distance among paired genotypes revealed the uniqueness of the studied genotypes and substantial genetic variability to be exploited in groundnut breeding. Overall, the study selected the following genetically divergent genotypes: ICGV 13189, ICGV 95111, ICGV 14421, and ICGV 171007, useful to develop breeding and mapping populations in groundnut improvement programs.

## Supporting information

S1 Table Mean values for 13 phenotypic traits of 100 groundnut genotypes under drought-stressed condition. (DOCX)

S2 Table Mean values for 13 phenotypic traits of 100 groundnut genotypes under non-stressed condition. (DOCX)

S3 Table Summary of 16, 363 SNP markers used genotyping 99 groundnut genotypes. (XLSX)

S4 Table Pairwise genetic distance matrix among 99 groundnut genotypes. (XLSX)

S5 Table Inferred ancestry of individuals and degree of admixture among 99 groundnut genotypes. (DOCX)

S6 Table Phenotypic distance matrix among 99 groundnut genotypes using gower’s distance matrix. (CSV)

S7 Table Genotypic dissimilarity matrix among 99 groundnut genotypes based on Jaccard’s Coefficient. (CSV)
