# Supplementary material for: Assessment of the genetic diversity and population structure of groundnut germplasm collections using phenotypic traits and SNP markers: Implications for drought tolerance breeding
Source: PLoS One. 2021 Nov 17;16(11):e0259883. doi: 10.1371/journal.pone.0259883 (PMC8598071; doi:10.1371/journal.pone.0259883)
Supplement: S1 Table — (DOCX) [file pone.0259883.s001.docx]

S1 Table. Mean values for 13 quantitative traits of 100 groundnut genotypes evaluated under drought-stressed condition in 2018/19 and 2019/20 post-rainy seasons.

| Sr. No | Genotype | DF | PH | PB | SCMR | LRWC | SLA | HAULM | POD | TBM | HI | SHP | HSW | SY |
| --- | --- | --- | --- | --- | --- | --- | --- | --- | --- | --- | --- | --- | --- | --- |
| 1 | ICGV 16667 | 50.00 | 16.70 | 8.15 | 49.64 | 56.49 | 121.80 | 20.79 | 3.74 | 24.53 | 14.67 | 48.01 | 18.79 | 1.79 |
| 2 | ICGV 93128 | 50.25 | 11.90 | 7.38 | 49.40 | 54.22 | 107.55 | 19.80 | 2.88 | 22.68 | 11.97 | 48.71 | 21.44 | 1.40 |
| 3 | ICGV 95066 | 50.25 | 13.73 | 6.65 | 47.68 | 57.51 | 123.73 | 17.88 | 5.64 | 23.51 | 21.71 | 44.73 | 24.70 | 2.52 |
| 4 | ICGV 96174 | 48.75 | 14.53 | 7.95 | 46.63 | 54.18 | 122.37 | 17.08 | 2.97 | 20.02 | 12.01 | 51.59 | 20.70 | 1.53 |
| 5 | ICGV 97087 | 50.00 | 15.43 | 7.15 | 43.19 | 70.87 | 129.17 | 20.03 | 4.79 | 24.82 | 16.63 | 50.45 | 24.89 | 2.42 |
| 6 | ICGV 98077 | 49.50 | 13.30 | 7.55 | 46.44 | 44.67 | 136.63 | 22.50 | 3.18 | 25.68 | 11.90 | 48.73 | 20.87 | 1.55 |
| 7 | ICGV 01279 | 49.75 | 13.80 | 8.65 | 46.94 | 59.27 | 128.87 | 20.62 | 3.25 | 23.87 | 12.79 | 54.89 | 24.06 | 1.78 |
| 8 | ICGV 03042 | 49.50 | 13.78 | 8.93 | 45.90 | 58.49 | 118.84 | 18.94 | 5.86 | 24.80 | 22.48 | 54.84 | 22.49 | 3.21 |
| 9 | ICGV 06039 | 51.00 | 12.18 | 7.23 | 47.35 | 55.70 | 117.72 | 13.98 | 5.69 | 19.67 | 26.44 | 50.98 | 20.45 | 2.90 |
| 10 | ICGV 06040 | 50.50 | 15.98 | 8.28 | 52.70 | 54.86 | 123.83 | 20.49 | 8.03 | 28.53 | 25.66 | 52.41 | 24.84 | 4.21 |
| 11 | ICGV 07010 | 49.25 | 13.95 | 8.45 | 45.93 | 50.41 | 138.04 | 20.51 | 4.92 | 25.43 | 16.49 | 45.99 | 23.47 | 2.26 |
| 12 | ICGV 10143 | 49.00 | 12.90 | 7.88 | 47.11 | 60.73 | 136.04 | 16.31 | 6.21 | 22.52 | 25.04 | 61.28 | 25.64 | 3.81 |
| 13 | ICGV 11422 | 50.25 | 13.03 | 8.45 | 48.08 | 48.51 | 115.07 | 21.99 | 2.30 | 24.29 | 10.03 | 43.80 | 19.35 | 1.01 |
| 14 | ICGV 11396 | 49.00 | 12.05 | 7.13 | 49.61 | 57.12 | 138.15 | 20.29 | 2.83 | 23.12 | 12.85 | 45.21 | 18.77 | 1.28 |
| 15 | ICGV 11418 | 50.50 | 14.03 | 7.23 | 46.48 | 56.92 | 126.52 | 20.49 | 2.88 | 23.37 | 11.55 | 51.65 | 20.74 | 1.49 |
| 16 | ICGV 91223 | 49.75 | 15.15 | 7.48 | 48.55 | 62.33 | 125.64 | 13.91 | 2.97 | 16.89 | 16.68 | 49.50 | 23.36 | 1.47 |
| 17 | ICGV 94118 | 49.75 | 15.45 | 6.60 | 43.99 | 60.48 | 115.79 | 14.55 | 4.96 | 19.51 | 21.88 | 52.34 | 23.67 | 2.60 |
| 18 | ICGV 99019 | 50.50 | 15.00 | 8.35 | 50.23 | 48.08 | 117.11 | 20.83 | 5.74 | 26.57 | 20.60 | 47.62 | 20.59 | 2.74 |
| 19 | ICGV 00162 | 49.50 | 16.18 | 8.23 | 43.60 | 47.33 | 120.30 | 17.12 | 3.86 | 20.98 | 17.77 | 50.21 | 21.34 | 1.94 |
| 20 | ICGV 00211 | 50.00 | 15.08 | 8.05 | 47.32 | 51.99 | 141.77 | 17.09 | 4.37 | 21.47 | 18.33 | 51.18 | 27.85 | 2.24 |
| 21 | ICGV 00187 | 49.25 | 14.50 | 7.33 | 53.56 | 63.82 | 113.56 | 17.10 | 4.97 | 22.06 | 22.65 | 46.24 | 19.47 | 2.30 |
| 22 | ICGV 00213 | 49.75 | 14.68 | 6.05 | 55.21 | 48.30 | 115.70 | 16.84 | 4.70 | 21.54 | 20.61 | 47.30 | 19.60 | 2.22 |
| 23 | ICGV 06146 | 50.50 | 15.88 | 7.75 | 40.87 | 57.57 | 115.01 | 14.48 | 5.51 | 19.99 | 24.83 | 54.96 | 23.06 | 3.03 |
| 24 | ICGV 07120 | 50.50 | 12.88 | 7.85 | 44.89 | 50.07 | 135.78 | 21.60 | 6.46 | 28.05 | 22.27 | 46.52 | 25.29 | 3.00 |
| 25 | ICGV 10178 | 49.75 | 15.68 | 7.53 | 43.12 | 48.50 | 129.83 | 23.01 | 7.12 | 30.13 | 22.04 | 55.99 | 24.03 | 3.99 |
| 26 | ICGV 11380 | 50.75 | 14.08 | 8.70 | 44.67 | 58.16 | 113.68 | 12.98 | 6.22 | 19.20 | 28.96 | 54.49 | 24.83 | 3.39 |
| 27 | ICGV 14001 | 50.50 | 14.98 | 7.68 | 42.56 | 57.87 | 115.47 | 17.17 | 5.40 | 22.57 | 21.83 | 52.71 | 21.21 | 2.85 |
| 28 | ICGV 14030 | 50.00 | 12.78 | 8.05 | 45.97 | 58.85 | 116.17 | 13.77 | 3.45 | 17.22 | 19.03 | 54.09 | 20.63 | 1.87 |
| 29 | ICGV 86015 | 50.00 | 11.35 | 8.25 | 49.26 | 56.82 | 120.28 | 13.90 | 3.87 | 17.77 | 20.51 | 53.48 | 25.29 | 2.07 |
| 30 | ICGV 93260 | 49.25 | 16.43 | 8.43 | 44.68 | 62.92 | 125.35 | 11.33 | 4.14 | 15.47 | 27.07 | 51.35 | 26.01 | 2.13 |
| 31 | ICGV 93261 | 49.00 | 13.65 | 7.43 | 47.17 | 48.35 | 114.21 | 14.30 | 5.28 | 19.58 | 27.24 | 49.53 | 19.55 | 2.61 |
| 32 | ICGV 92121 | 49.75 | 14.30 | 7.30 | 46.46 | 52.30 | 116.31 | 22.95 | 5.57 | 28.52 | 17.07 | 55.01 | 20.44 | 3.06 |
| 33 | ICGV 99241 | 49.50 | 15.23 | 8.30 | 45.45 | 52.05 | 135.45 | 23.26 | 6.50 | 29.76 | 20.78 | 52.47 | 22.96 | 3.41 |
| Table S1. Continued. | | | | | | | | | | | | | | |
| Sr. No | Genotype | DF | PH | PB | SCMR | LRWC | SLA | HAULM | POD | TBM | HI | SHP | HSW | SY |
| 34 | ICGV 00351 | 50.25 | 16.70 | 6.98 | 43.61 | 54.47 | 129.18 | 19.44 | 4.14 | 23.58 | 16.21 | 53.80 | 21.00 | 2.23 |
| 35 | ICGV 01260 | 50.50 | 13.45 | 6.70 | 50.17 | 52.65 | 121.62 | 28.56 | 8.57 | 37.13 | 20.74 | 48.68 | 26.04 | 4.17 |
| 36 | ICGV 01265 | 49.75 | 13.55 | 7.38 | 50.90 | 58.21 | 121.24 | 18.09 | 5.50 | 23.59 | 19.77 | 50.61 | 25.02 | 2.78 |
| 37 | ICGV 13200 | 50.25 | 14.88 | 6.45 | 44.30 | 58.16 | 121.13 | 15.19 | 7.04 | 22.22 | 26.74 | 49.59 | 20.78 | 3.49 |
| 38 | ICGV 07220 | 50.50 | 12.48 | 9.18 | 45.76 | 54.65 | 121.35 | 13.16 | 2.35 | 15.51 | 13.00 | 46.47 | 22.57 | 1.09 |
| 39 | ICGV 07222 | 50.00 | 12.25 | 7.85 | 45.13 | 61.16 | 124.53 | 18.61 | 7.19 | 25.80 | 25.21 | 54.49 | 26.51 | 3.92 |
| 40 | ICGV 13317 | 50.25 | 11.63 | 7.88 | 46.87 | 58.74 | 119.32 | 17.95 | 4.65 | 22.60 | 20.78 | 55.55 | 24.88 | 2.58 |
| 41 | ICGV 13254 | 48.75 | 13.23 | 7.75 | 52.38 | 54.34 | 119.00 | 17.60 | 3.87 | 21.47 | 17.49 | 50.14 | 19.79 | 1.94 |
| 42 | ICGV 181026 | 50.75 | 12.75 | 7.65 | 49.39 | 47.98 | 130.97 | 16.21 | 3.77 | 19.98 | 16.98 | 50.46 | 20.46 | 1.90 |
| 43 | ICGV 15073 | 48.75 | 16.33 | 8.75 | 47.85 | 53.43 | 112.82 | 18.07 | 5.05 | 23.12 | 19.86 | 52.34 | 20.73 | 2.64 |
| 44 | ICGV 15074 | 50.00 | 14.98 | 8.50 | 49.73 | 53.74 | 116.71 | 15.50 | 5.20 | 20.70 | 22.67 | 49.04 | 20.02 | 2.55 |
| 45 | ICGV 15083 | 50.25 | 15.93 | 9.60 | 47.15 | 55.14 | 118.43 | 18.03 | 6.74 | 24.77 | 25.66 | 48.91 | 22.83 | 3.30 |
| 46 | ICGV 15019 | 49.50 | 13.75 | 7.50 | 46.00 | 45.22 | 124.05 | 16.16 | 5.73 | 21.90 | 23.14 | 51.04 | 23.09 | 2.93 |
| 47 | ICGV 06420 | 51.50 | 14.28 | 8.05 | 47.75 | 74.07 | 141.75 | 18.51 | 4.50 | 23.01 | 18.69 | 56.58 | 28.06 | 2.54 |
| 48 | ICGV 05155 | 50.50 | 13.85 | 8.73 | 48.48 | 45.62 | 126.23 | 20.51 | 4.45 | 24.96 | 18.26 | 46.85 | 18.18 | 2.08 |
| 49 | ICGV 16688 | 48.75 | 15.23 | 8.75 | 49.25 | 60.98 | 140.61 | 20.87 | 4.80 | 25.67 | 17.36 | 51.51 | 24.67 | 2.47 |
| 50 | ICGV 03043 | 50.50 | 14.23 | 8.25 | 44.34 | 59.82 | 125.91 | 19.17 | 4.64 | 23.81 | 18.57 | 54.22 | 24.10 | 2.52 |
| 51 | ICGV 00350 | 47.75 | 13.13 | 7.35 | 42.03 | 53.10 | 129.92 | 16.89 | 4.60 | 21.49 | 19.37 | 52.59 | 25.50 | 2.42 |
| 52 | ICGV 86590 | 50.00 | 14.10 | 5.68 | 46.01 | 67.49 | 126.04 | 17.93 | 4.61 | 22.54 | 17.26 | 53.75 | 24.23 | 2.48 |
| 53 | ICGV 02266 | 49.25 | 13.53 | 7.55 | 50.30 | 58.12 | 134.38 | 14.61 | 6.13 | 20.74 | 27.01 | 48.77 | 32.53 | 2.99 |
| 54 | ICGV 13189 | 49.50 | 13.85 | 5.18 | 43.48 | 58.23 | 129.67 | 12.47 | 5.11 | 17.58 | 26.76 | 57.48 | 27.03 | 2.94 |
| 55 | ICGV 13207 | 49.50 | 11.25 | 8.58 | 46.65 | 59.73 | 110.68 | 9.74 | 4.98 | 14.72 | 29.87 | 52.43 | 23.30 | 2.61 |
| 56 | ICGV 14421 | 49.50 | 11.95 | 6.43 | 43.88 | 62.35 | 118.59 | 14.21 | 6.35 | 20.56 | 28.96 | 54.92 | 24.08 | 3.49 |
| 57 | ICGV 13219 | 48.75 | 13.08 | 4.70 | 43.65 | 59.10 | 128.94 | 13.62 | 4.26 | 17.88 | 21.35 | 52.76 | 23.76 | 2.25 |
| 58 | GPBD 4 | 49.25 | 13.50 | 6.28 | 41.91 | 46.18 | 125.53 | 12.73 | 3.68 | 16.40 | 19.67 | 48.31 | 18.99 | 1.78 |
| 59 | ICGV 86031 | 49.50 | 14.78 | 7.40 | 50.05 | 52.58 | 108.92 | 13.66 | 3.70 | 17.36 | 20.11 | 50.57 | 20.89 | 1.87 |
| 60 | ICGV 16686 | 50.75 | 16.30 | 7.38 | 50.65 | 47.06 | 131.13 | 21.28 | 4.94 | 26.22 | 16.56 | 47.08 | 18.76 | 2.33 |
| 61 | ICGV 16005 | 50.00 | 13.85 | 6.03 | 49.20 | 56.60 | 122.12 | 17.10 | 3.73 | 20.83 | 17.58 | 50.69 | 20.57 | 1.89 |
| 62 | ICGV 171013 | 49.00 | 13.45 | 7.60 | 54.28 | 59.28 | 121.61 | 17.17 | 5.50 | 22.67 | 22.33 | 55.23 | 24.45 | 3.04 |
| 63 | ICGV 171026 | 49.75 | 12.28 | 7.78 | 49.30 | 53.86 | 122.64 | 20.13 | 5.13 | 25.27 | 18.32 | 53.30 | 21.17 | 2.74 |
| 64 | ICGV 171039 | 48.25 | 15.53 | 7.28 | 51.32 | 61.48 | 118.64 | 15.89 | 5.81 | 21.71 | 23.18 | 49.36 | 22.71 | 2.87 |
| 65 | ICGV 171046 | 50.25 | 13.78 | 8.03 | 45.68 | 55.41 | 117.42 | 14.97 | 6.75 | 21.72 | 26.64 | 52.16 | 29.16 | 3.52 |
| 66 | ICGV 181017 | 50.00 | 12.15 | 7.98 | 40.73 | 65.06 | 115.73 | 20.20 | 5.88 | 26.08 | 19.78 | 51.08 | 26.20 | 3.00 |
| 67 | ICGV 181063 | 49.00 | 14.50 | 8.60 | 48.97 | 67.87 | 123.98 | 19.15 | 3.56 | 22.71 | 14.12 | 49.18 | 26.92 | 1.75 |
| Table S1. Continued. | | | | | | | | | | | | | | |
| Sr. No | Genotype | DF | PH | PB | SCMR | LRWC | SLA | HAULM | POD | TBM | HI | SHP | HSW | SY |
| 68 | ICGV 98412 | 49.25 | 17.98 | 7.95 | 52.31 | 48.01 | 133.98 | 21.08 | 5.56 | 26.64 | 17.53 | 46.65 | 26.22 | 2.59 |
| 69 | ICGV 181489 | 50.25 | 15.38 | 7.85 | 42.57 | 57.89 | 136.94 | 22.21 | 3.63 | 25.84 | 12.58 | 44.74 | 23.11 | 1.62 |
| 70 | ICGV 181490 | 50.25 | 14.00 | 6.65 | 50.26 | 71.68 | 129.11 | 16.51 | 5.42 | 21.93 | 20.91 | 60.85 | 26.99 | 3.30 |
| 71 | ICGV 92054 | 51.75 | 14.55 | 8.03 | 48.64 | 58.43 | 122.08 | 19.73 | 3.88 | 23.62 | 16.78 | 51.33 | 22.40 | 1.99 |
| 72 | ICGV 93162 | 51.00 | 15.83 | 9.10 | 44.86 | 51.48 | 133.84 | 23.72 | 3.96 | 27.68 | 14.48 | 47.34 | 26.55 | 1.88 |
| 73 | ICGV 95111 | 52.00 | 13.78 | 9.45 | 46.68 | 45.78 | 123.14 | 21.15 | 5.27 | 23.92 | 21.20 | 50.12 | 21.43 | 2.64 |
| 74 | ICGV 96165 | 51.50 | 11.50 | 9.83 | 45.93 | 71.47 | 107.41 | 26.72 | 4.06 | 30.78 | 12.55 | 50.95 | 21.28 | 2.07 |
| 75 | ICGV 97115 | 51.75 | 13.18 | 8.13 | 51.31 | 57.86 | 129.50 | 18.44 | 4.05 | 22.49 | 16.83 | 48.84 | 22.35 | 1.98 |
| 76 | ICGV 98184 | 50.25 | 13.25 | 7.33 | 45.73 | 56.56 | 127.76 | 22.37 | 4.61 | 26.98 | 17.24 | 48.53 | 23.43 | 2.24 |
| 77 | ICGV 01491 | 51.00 | 16.95 | 7.83 | 47.98 | 48.08 | 124.03 | 19.72 | 3.40 | 23.12 | 15.20 | 51.01 | 20.23 | 1.74 |
| 78 | ICGV 03287 | 51.50 | 15.50 | 9.30 | 48.00 | 51.81 | 125.78 | 18.41 | 4.20 | 22.61 | 17.21 | 48.07 | 18.73 | 2.02 |
| 79 | ICGV 05057 | 51.75 | 11.93 | 10.38 | 53.45 | 55.29 | 118.34 | 18.94 | 3.79 | 22.74 | 17.30 | 48.26 | 24.07 | 1.83 |
| 80 | ICGV 06175 | 50.00 | 14.35 | 8.18 | 47.10 | 54.32 | 130.16 | 18.66 | 7.51 | 26.17 | 27.76 | 52.78 | 25.24 | 3.96 |
| 81 | ICGV 00064 | 51.50 | 13.40 | 6.85 | 42.99 | 54.35 | 133.74 | 23.07 | 5.17 | 28.25 | 16.61 | 51.91 | 21.36 | 2.69 |
| 82 | ICGV 00246 | 51.00 | 15.70 | 8.00 | 45.17 | 65.42 | 130.07 | 19.36 | 4.58 | 23.94 | 18.37 | 48.45 | 22.21 | 2.22 |
| 83 | ICGV 97150 | 51.00 | 14.10 | 9.40 | 48.80 | 58.82 | 130.11 | 20.29 | 2.51 | 22.79 | 10.62 | 45.70 | 25.42 | 1.15 |
| 84 | ICGV 98385 | 51.50 | 15.63 | 9.95 | 47.40 | 61.53 | 124.64 | 20.11 | 2.23 | 22.33 | 10.18 | 45.73 | 21.62 | 1.02 |
| 85 | ICGV 96266 | 51.50 | 13.63 | 9.73 | 51.19 | 66.40 | 125.92 | 24.59 | 3.27 | 27.86 | 11.83 | 48.86 | 22.28 | 1.60 |
| 86 | ICGV 14224 | 50.25 | 12.65 | 8.98 | 54.17 | 51.29 | 126.27 | 21.35 | 5.02 | 26.37 | 18.64 | 49.26 | 23.00 | 2.47 |
| 87 | ICGV 14232 | 51.25 | 14.88 | 8.23 | 49.18 | 51.71 | 127.97 | 23.85 | 5.29 | 29.14 | 17.02 | 52.40 | 21.76 | 2.77 |
| 88 | ICGV 07262 | 51.00 | 15.38 | 9.35 | 45.63 | 61.03 | 120.27 | 18.59 | 4.25 | 22.84 | 18.84 | 50.42 | 22.73 | 2.14 |
| 89 | ICGV 07247 | 51.25 | 11.98 | 7.93 | 47.01 | 56.55 | 120.97 | 16.84 | 3.93 | 20.77 | 16.90 | 49.45 | 23.92 | 1.94 |
| 90 | ICGV 10371 | 50.25 | 15.40 | 7.93 | 46.82 | 53.04 | 129.02 | 17.90 | 4.41 | 22.31 | 19.28 | 49.01 | 22.32 | 2.16 |
| 91 | ICGV 10373 | 52.50 | 13.73 | 8.98 | 47.87 | 45.10 | 117.89 | 21.37 | 6.57 | 27.93 | 21.64 | 60.11 | 17.98 | 3.95 |
| 92 | ICGV 10379 | 51.50 | 13.73 | 9.03 | 49.20 | 59.72 | 122.93 | 21.01 | 5.08 | 26.08 | 19.46 | 49.60 | 19.52 | 2.52 |
| 93 | ICGV 15094 | 52.00 | 17.15 | 8.50 | 48.62 | 65.42 | 133.51 | 19.30 | 2.90 | 22.20 | 12.63 | 47.97 | 20.07 | 1.39 |
| 94 | ICGV 87846 | 51.75 | 15.73 | 10.05 | 50.86 | 59.50 | 123.57 | 19.27 | 4.71 | 23.98 | 18.70 | 48.82 | 23.63 | 2.30 |
| 95 | ICGV 86699 | 50.50 | 13.58 | 8.35 | 46.36 | 52.63 | 117.17 | 20.25 | 4.15 | 24.40 | 16.90 | 45.04 | 25.83 | 1.87 |
| 96 | GG 20 | 52.25 | 12.43 | 9.10 | 49.53 | 62.35 | 125.74 | 22.05 | 3.74 | 25.80 | 14.27 | 50.89 | 24.24 | 1.91 |
| 97 | ICGV 171007 | 49.75 | 13.70 | 8.95 | 51.42 | 57.24 | 126.25 | 17.93 | 2.95 | 20.88 | 13.83 | 48.67 | 27.08 | 1.44 |
| 98 | ICGV 171027 | 51.50 | 15.38 | 9.25 | 43.65 | 59.78 | 117.09 | 25.21 | 4.24 | 29.45 | 14.46 | 50.88 | 20.14 | 2.16 |
| 99 | ICGV 181006 | 51.25 | 13.30 | 10.05 | 45.80 | 53.94 | 125.30 | 18.97 | 2.74 | 21.71 | 12.68 | 54.57 | 23.39 | 1.49 |
| 100 | ICGV 181033 | 51.25 | 13.63 | 10.58 | 45.98 | 52.38 | 125.89 | 20.61 | 6.24 | 26.85 | 20.96 | 51.85 | 23.30 | 3.24 |
|  |  |  |  |  |  |  |  |  |  |  |  |  |  |  |
| Table S1. Continued. | | | | | | | | | | | | | | |
|  | | DF | PH | PB | SCMR | LRWC | SLA | HAULM | POD | TBM | HI | SHP | HSW | SY |
| CV (%) | | 1.99 | 14.61 | 16.19 | 19.56 | 25.98 | 10.31 | 18.20 | 27.17 | 15.61 | 21.35 | 7.59 | 19.15 | 33.70 |
| SE | | 1.00 | 2.06 | 1.29 | 9.35 | 14.64 | 12.81 | 3.38 | 1.28 | 3.69 | 4.04 | 6.95 | 4.39 | 0.84 |
| LSD (5%) | | 1.40 | 2.88 | 1.81 | 13.06 | 20.44 | 17.89 | 4.73 | 1.79 | 5.15 | 5.64 | 9.70 | 6.13 | 1.17 |

DF=days to 50% flowering, PH=plant height, PB=number of primary branches per plant, SCMR=SPAD chlorophyll meter reading, LRWC=leaf relative water content, SLA=specific leaf area (cm^2^ g^-1^), HAULM=haulm weight (g plant^-1^), SHP=shelling percentage, HSW=hundred seed weight(g), PY=pod yield (g plant^-1^ ), HI=harvest index (%), TBM=total biomass production ( g plant^-1^ ) (g), SY=seed (g plant^-1^).
