# Supplementary material for: Assessment of the genetic diversity and population structure of groundnut germplasm collections using phenotypic traits and SNP markers: Implications for drought tolerance breeding
Source: PLoS One. 2021 Nov 17;16(11):e0259883. doi: 10.1371/journal.pone.0259883 (PMC8598071; doi:10.1371/journal.pone.0259883)
Supplement: S2 Table — (DOCX) [file pone.0259883.s002.docx]

S2 Table. Mean values for 13 phenotypic traits of 100 groundnut genotypes evaluated under non-stressed condition in 2018/19 and 2019/20 post-rainy seasons.

| Sr. No. | Genotype | DF | PH | PB | SCMR | LRWC | SLA | HAULM | POD | TBM | HI | SHP | HSW | SY |
| --- | --- | --- | --- | --- | --- | --- | --- | --- | --- | --- | --- | --- | --- | --- |
| 1 | ICGV 16667 | 50.75 | 17.55 | 8.15 | 49.45 | 61.05 | 129.64 | 28.23 | 11.14 | 39.37 | 28.76 | 48.03 | 21.88 | 5.35 |
| 2 | ICGV 93128 | 50.25 | 13.85 | 6.15 | 51.64 | 65.88 | 137.97 | 22.71 | 10.44 | 33.15 | 31.97 | 60.85 | 27.69 | 6.36 |
| 3 | ICGV 95066 | 50.25 | 20.4 | 8.6 | 48.27 | 66.23 | 117.22 | 27.55 | 10.84 | 38.40 | 28.70 | 47.88 | 21.98 | 5.19 |
| 4 | ICGV 96174 | 48.75 | 20.45 | 8.05 | 46.10 | 64.08 | 124.81 | 28.86 | 8.34 | 37.20 | 23.57 | 55.03 | 28.43 | 4.59 |
| 5 | ICGV 97087 | 50.25 | 18.8 | 9.85 | 42.48 | 59.67 | 132.27 | 32.39 | 9.34 | 41.73 | 24.20 | 48.61 | 29.23 | 4.54 |
| 6 | ICGV 98077 | 50.5 | 16.9 | 8.05 | 49.46 | 72.66 | 135.84 | 27.86 | 8.25 | 36.11 | 23.30 | 56.95 | 31.49 | 4.70 |
| 7 | ICGV 01279 | 51 | 16.45 | 9.75 | 47.60 | 68.35 | 132.09 | 25.88 | 10.66 | 36.54 | 29.72 | 60.63 | 31.65 | 6.46 |
| 8 | ICGV 03042 | 50.25 | 17.5 | 7.95 | 50.04 | 71.71 | 133.75 | 23.13 | 14.64 | 37.77 | 38.71 | 62.36 | 30.77 | 9.13 |
| 9 | ICGV 06039 | 52 | 20.05 | 7.55 | 46.63 | 67.19 | 120.43 | 20.62 | 14.95 | 35.57 | 42.76 | 60.64 | 27.96 | 9.07 |
| 10 | ICGV 06040 | 49.25 | 17.15 | 10.1 | 50.57 | 70.70 | 127.52 | 23.06 | 14.13 | 37.19 | 38.02 | 58.88 | 25.86 | 8.32 |
| 11 | ICGV 07010 | 50 | 21.15 | 10.1 | 45.28 | 68.00 | 140.56 | 23.56 | 12.86 | 36.42 | 36.02 | 54.29 | 32.45 | 6.98 |
| 12 | ICGV 10143 | 49.75 | 20.2 | 9.65 | 45.93 | 81.11 | 142.00 | 23.53 | 15.49 | 39.03 | 40.45 | 67.88 | 29.37 | 10.52 |
| 13 | ICGV 11422 | 49.75 | 19.15 | 9.05 | 46.40 | 63.88 | 131.65 | 30.17 | 8.74 | 38.91 | 22.69 | 52.21 | 27.18 | 4.56 |
| 14 | ICGV 11396 | 51 | 19.25 | 8.2 | 50.70 | 77.07 | 138.28 | 27.28 | 13.20 | 40.48 | 33.55 | 56.94 | 24.78 | 7.52 |
| 15 | ICGV 11418 | 50.75 | 19.4 | 8.9 | 46.49 | 53.57 | 136.75 | 30.26 | 10.61 | 40.87 | 27.51 | 50.71 | 26.73 | 5.38 |
| 16 | ICGV 91223 | 51 | 20.95 | 9.35 | 39.20 | 77.14 | 155.83 | 24.55 | 9.51 | 34.06 | 30.34 | 57.10 | 28.89 | 5.43 |
| 17 | ICGV 94118 | 49.5 | 17.9 | 8.3 | 49.30 | 61.85 | 109.38 | 27.90 | 13.05 | 40.94 | 30.54 | 50.99 | 21.93 | 6.65 |
| 18 | ICGV 99019 | 50.25 | 18.45 | 7.45 | 50.95 | 80.61 | 127.29 | 28.53 | 11.50 | 40.04 | 28.64 | 62.39 | 27.89 | 7.18 |
| 19 | ICGV 00162 | 50.25 | 23 | 9.5 | 46.97 | 53.02 | 138.52 | 25.43 | 9.00 | 34.43 | 25.85 | 53.79 | 22.50 | 4.84 |
| 20 | ICGV 00211 | 50 | 17.05 | 7.55 | 44.40 | 75.86 | 142.81 | 31.56 | 12.69 | 44.25 | 30.86 | 55.96 | 24.11 | 7.10 |
| 21 | ICGV 00187 | 49.5 | 18.7 | 5.9 | 52.89 | 65.34 | 119.65 | 22.30 | 8.80 | 31.10 | 28.30 | 52.97 | 26.15 | 4.66 |
| 22 | ICGV 00213 | 49.25 | 19.2 | 7.2 | 50.85 | 75.84 | 130.65 | 24.51 | 11.11 | 35.63 | 33.72 | 56.70 | 22.18 | 6.30 |
| 23 | ICGV 06146 | 49.75 | 20.45 | 9.45 | 43.04 | 66.14 | 123.16 | 17.22 | 10.25 | 27.48 | 37.88 | 52.39 | 29.47 | 5.37 |
| 24 | ICGV 07120 | 52.25 | 13.85 | 9.1 | 50.02 | 66.45 | 142.82 | 22.35 | 8.52 | 30.86 | 27.86 | 57.19 | 36.09 | 4.87 |
| 25 | ICGV 10178 | 49.75 | 19.95 | 10.1 | 46.18 | 53.11 | 127.15 | 27.13 | 10.79 | 37.92 | 30.88 | 49.32 | 26.65 | 5.32 |
| 26 | ICGV 11380 | 52 | 21.95 | 7.65 | 46.57 | 68.77 | 123.36 | 20.33 | 13.08 | 33.41 | 39.78 | 61.62 | 24.14 | 8.06 |
| 27 | ICGV 14001 | 50.25 | 18.75 | 8.8 | 43.10 | 63.57 | 119.75 | 22.78 | 15.09 | 37.87 | 42.56 | 56.12 | 22.35 | 8.47 |
| 28 | ICGV 14030 | 50.5 | 19.8 | 9.6 | 47.34 | 72.07 | 139.04 | 20.06 | 12.26 | 32.32 | 38.12 | 64.24 | 30.25 | 7.88 |
| 29 | ICGV 86015 | 48.75 | 17.2 | 7.5 | 49.25 | 61.78 | 123.44 | 20.81 | 12.59 | 33.40 | 38.78 | 55.51 | 23.38 | 6.99 |
| 30 | ICGV 93260 | 50 | 15.85 | 8.75 | 45.77 | 73.75 | 128.73 | 23.85 | 14.11 | 37.96 | 36.82 | 59.10 | 26.85 | 8.34 |
| 31 | ICGV 93261 | 49.5 | 19.05 | 7.3 | 51.04 | 57.68 | 118.61 | 18.63 | 14.00 | 32.63 | 41.69 | 52.03 | 23.15 | 7.28 |
| 32 | ICGV 92121 | 50 | 18.65 | 8.7 | 43.73 | 71.18 | 127.75 | 26.65 | 11.05 | 37.70 | 28.47 | 52.11 | 26.99 | 5.76 |
| 33 | ICGV 99241 | 51 | 19.85 | 9.25 | 42.40 | 69.91 | 130.85 | 30.33 | 11.94 | 42.26 | 28.68 | 56.17 | 29.55 | 6.71 |
| Table S2. Continued. | | | | | | | | | | | | | | |
| Sr. No. | Genotype | DF | PH | PB | SCMR | LRWC | SLA | HAULM | POD | TBM | HI | SHP | HSW | SY |
| 34 | ICGV 00351 | 50.25 | 20.3 | 6.05 | 42.57 | 73.93 | 151.97 | 21.58 | 10.47 | 32.05 | 33.93 | 56.12 | 29.25 | 5.88 |
| 35 | ICGV 01260 | 50.75 | 17.65 | 7.5 | 48.30 | 65.36 | 147.81 | 28.07 | 11.68 | 39.75 | 29.65 | 54.45 | 36.10 | 6.36 |
| 36 | ICGV 01265 | 50.5 | 16.55 | 8.15 | 54.26 | 64.66 | 116.20 | 22.20 | 9.35 | 31.55 | 30.17 | 64.06 | 34.29 | 5.99 |
| 37 | ICGV 13200 | 49.5 | 18.9 | 8.6 | 43.80 | 57.95 | 128.37 | 15.77 | 11.50 | 27.27 | 40.87 | 53.46 | 20.51 | 6.15 |
| 38 | ICGV 07220 | 51 | 15.65 | 11.4 | 47.13 | 79.73 | 144.16 | 19.55 | 12.37 | 31.92 | 38.30 | 58.17 | 25.75 | 7.20 |
| 39 | ICGV 07222 | 50.75 | 14.5 | 10.85 | 43.46 | 75.88 | 130.05 | 18.45 | 15.93 | 34.38 | 47.21 | 60.90 | 29.88 | 9.70 |
| 40 | ICGV 13317 | 50.5 | 15.2 | 8.55 | 46.66 | 74.91 | 129.26 | 22.97 | 13.38 | 36.35 | 37.14 | 64.25 | 32.71 | 8.59 |
| 41 | ICGV 13254 | 50.25 | 19.45 | 7.75 | 52.09 | 69.98 | 128.96 | 29.13 | 14.39 | 43.52 | 31.78 | 53.37 | 22.73 | 7.68 |
| 42 | ICGV 181026 | 50.5 | 18.95 | 9.35 | 44.48 | 70.96 | 130.00 | 27.89 | 12.34 | 40.23 | 30.82 | 53.09 | 24.94 | 6.55 |
| 43 | ICGV 15073 | 50 | 17.2 | 9.9 | 46.88 | 57.38 | 130.70 | 27.40 | 9.94 | 37.34 | 26.77 | 49.82 | 27.05 | 4.95 |
| 44 | ICGV 15074 | 50.25 | 22.3 | 9.55 | 48.21 | 64.70 | 132.53 | 26.50 | 13.03 | 39.52 | 32.52 | 55.28 | 27.10 | 7.20 |
| 45 | ICGV 15083 | 50.75 | 19.55 | 9.2 | 51.10 | 55.26 | 127.24 | 29.24 | 14.69 | 43.93 | 33.79 | 51.79 | 28.09 | 7.61 |
| 46 | ICGV 15019 | 49.25 | 19.55 | 8.7 | 49.39 | 80.59 | 132.09 | 28.13 | 10.11 | 38.25 | 27.94 | 60.35 | 27.78 | 6.10 |
| 47 | ICGV 06420 | 49.75 | 20.15 | 8.85 | 53.56 | 74.95 | 130.72 | 29.14 | 12.50 | 41.64 | 30.56 | 53.03 | 22.68 | 6.63 |
| 48 | ICGV 05155 | 51.25 | 16.8 | 8.6 | 50.60 | 73.72 | 136.39 | 19.81 | 10.76 | 30.57 | 34.12 | 65.26 | 24.03 | 7.02 |
| 49 | ICGV 16688 | 49.25 | 21.8 | 8.6 | 49.65 | 66.72 | 126.86 | 28.48 | 12.44 | 40.92 | 30.99 | 53.83 | 25.28 | 6.70 |
| 50 | ICGV 03043 | 50 | 19.2 | 10 | 48.79 | 81.17 | 133.15 | 23.89 | 12.59 | 36.47 | 34.61 | 55.13 | 30.22 | 6.94 |
| 51 | ICGV 00350 | 49.25 | 19.35 | 6.55 | 48.16 | 55.98 | 136.44 | 25.93 | 14.68 | 40.61 | 35.91 | 52.65 | 26.04 | 7.73 |
| 52 | ICGV 86590 | 50.75 | 19.35 | 8.5 | 48.65 | 66.20 | 124.94 | 28.84 | 9.69 | 38.53 | 25.37 | 55.07 | 27.13 | 5.34 |
| 53 | ICGV 02266 | 50.5 | 17.25 | 9 | 47.37 | 62.48 | 132.10 | 24.41 | 12.76 | 37.16 | 35.59 | 49.16 | 27.73 | 6.27 |
| 54 | ICGV 13189 | 50.25 | 16.5 | 6.55 | 49.12 | 69.79 | 136.07 | 19.49 | 12.33 | 31.82 | 41.08 | 60.91 | 26.80 | 7.51 |
| 55 | ICGV 13207 | 50.25 | 12.35 | 6.1 | 45.07 | 58.95 | 131.33 | 12.97 | 10.43 | 23.41 | 44.40 | 54.10 | 19.70 | 5.65 |
| 56 | ICGV 14421 | 48 | 14.2 | 6.4 | 44.83 | 80.53 | 132.28 | 21.94 | 12.26 | 34.20 | 38.31 | 66.31 | 26.15 | 8.13 |
| 57 | ICGV 13219 | 49.5 | 12.95 | 4.7 | 46.53 | 71.32 | 128.95 | 15.82 | 7.64 | 23.46 | 34.00 | 52.81 | 24.43 | 4.03 |
| 58 | GPBD 4 | 50 | 19.1 | 6.7 | 44.63 | 81.34 | 134.39 | 22.53 | 7.30 | 29.84 | 25.45 | 63.78 | 28.92 | 4.66 |
| 59 | ICGV 86031 | 50 | 20.65 | 5.75 | 52.34 | 62.42 | 131.66 | 21.08 | 8.75 | 29.83 | 29.25 | 59.62 | 24.62 | 5.21 |
| 60 | ICGV 16686 | 49.75 | 20.75 | 8 | 49.03 | 76.50 | 133.21 | 27.29 | 10.42 | 37.72 | 29.20 | 54.54 | 23.60 | 5.68 |
| 61 | ICGV 16005 | 48.75 | 15 | 7.1 | 52.01 | 57.90 | 134.56 | 25.63 | 11.85 | 37.47 | 32.14 | 49.13 | 21.69 | 5.82 |
| 62 | ICGV 171013 | 50.5 | 13.2 | 7.1 | 57.39 | 75.93 | 143.11 | 16.90 | 10.29 | 27.18 | 40.80 | 57.48 | 35.70 | 5.91 |
| 63 | ICGV 171026 | 50.25 | 14.2 | 8 | 51.60 | 61.28 | 128.54 | 25.93 | 10.07 | 36.00 | 28.15 | 46.83 | 21.41 | 4.72 |
| 64 | ICGV 171039 | 49.75 | 22.75 | 8 | 49.57 | 75.33 | 138.81 | 22.78 | 11.62 | 34.40 | 34.43 | 56.36 | 29.03 | 6.55 |
| 65 | ICGV 171046 | 50.25 | 17.45 | 8.8 | 46.05 | 65.27 | 130.15 | 23.19 | 10.12 | 33.31 | 31.03 | 50.66 | 27.35 | 5.13 |
| 66 | ICGV 181017 | 51 | 20.25 | 9.5 | 41.29 | 77.99 | 121.57 | 31.13 | 10.74 | 41.87 | 26.75 | 50.95 | 23.80 | 5.47 |
| 67 | ICGV 181063 | 50.5 | 26.2 | 8.45 | 48.47 | 63.05 | 144.04 | 29.41 | 6.97 | 36.37 | 18.91 | 42.94 | 26.05 | 2.99 |
| Table S2. Continued. | | | | | | | | | | | | | | |
| Sr. No. | Genotype | DF | PH | PB | SCMR | LRWC | SLA | HAULM | POD | TBM | HI | SHP | HSW | SY |
| 68 | ICGV 98412 | 50.75 | 24.1 | 10.75 | 51.08 | 72.96 | 137.41 | 24.92 | 16.21 | 41.13 | 40.79 | 55.70 | 36.22 | 9.03 |
| 69 | ICGV 181489 | 52 | 18.9 | 10.2 | 41.47 | 61.23 | 131.67 | 29.03 | 6.75 | 35.78 | 19.24 | 47.53 | 22.25 | 3.21 |
| 70 | ICGV 181490 | 49.5 | 18.95 | 8.85 | 47.62 | 70.17 | 128.60 | 20.12 | 10.43 | 30.56 | 34.77 | 60.54 | 29.34 | 6.32 |
| 71 | ICGV 92054 | 51 | 18.1 | 9.6 | 51.78 | 50.75 | 132.45 | 27.74 | 8.03 | 35.77 | 22.48 | 46.88 | 23.68 | 3.76 |
| 72 | ICGV 93162 | 50 | 20.35 | 9.55 | 44.73 | 73.16 | 133.94 | 27.06 | 8.47 | 35.53 | 24.46 | 59.28 | 28.66 | 5.02 |
| 73 | ICGV 95111 | 51.75 | 16.45 | 10.25 | 50.99 | 61.91 | 127.88 | 29.10 | 7.12 | 36.22 | 19.04 | 54.78 | 24.89 | 3.90 |
| 74 | ICGV 96165 | 50.75 | 15.4 | 7.95 | 50.99 | 80.45 | 137.85 | 25.20 | 7.68 | 32.88 | 23.89 | 59.04 | 30.31 | 4.53 |
| 75 | ICGV 97115 | 52.75 | 15.4 | 9.35 | 41.78 | 70.85 | 129.67 | 31.34 | 9.19 | 40.52 | 23.56 | 49.60 | 23.62 | 4.56 |
| 76 | ICGV 98184 | 51 | 16.2 | 9.95 | 49.32 | 78.45 | 125.48 | 23.78 | 7.87 | 31.65 | 24.77 | 61.95 | 30.21 | 4.88 |
| 77 | ICGV 01491 | 51.25 | 22.9 | 9 | 47.07 | 63.06 | 121.55 | 34.51 | 8.59 | 43.09 | 19.67 | 50.56 | 23.70 | 4.34 |
| 78 | ICGV 03287 | 51.5 | 22.7 | 9.25 | 50.19 | 76.84 | 124.12 | 25.12 | 8.52 | 33.64 | 24.69 | 64.10 | 26.22 | 5.46 |
| 79 | ICGV 05057 | 51 | 17.75 | 9.25 | 44.47 | 59.81 | 120.72 | 23.87 | 9.63 | 33.50 | 29.21 | 55.03 | 25.65 | 5.30 |
| 80 | ICGV 06175 | 50.75 | 20.8 | 10.85 | 49.63 | 78.37 | 132.57 | 25.35 | 12.18 | 37.53 | 32.15 | 62.90 | 25.25 | 7.66 |
| 81 | ICGV 00064 | 50.75 | 16.2 | 9.05 | 46.43 | 61.09 | 130.39 | 28.38 | 8.53 | 36.91 | 24.44 | 57.31 | 21.38 | 4.89 |
| 82 | ICGV 00246 | 50.5 | 23.35 | 8.15 | 48.27 | 76.87 | 125.89 | 24.94 | 8.23 | 33.17 | 24.45 | 57.01 | 23.88 | 4.69 |
| 83 | ICGV 97150 | 50.5 | 19.85 | 10.85 | 48.00 | 65.43 | 120.56 | 28.94 | 6.09 | 35.03 | 17.00 | 45.51 | 23.56 | 2.77 |
| 84 | ICGV 98385 | 50.75 | 19.8 | 13.15 | 45.33 | 78.78 | 131.08 | 30.16 | 6.57 | 36.73 | 18.48 | 58.08 | 28.68 | 3.81 |
| 85 | ICGV 96266 | 49.75 | 21.5 | 8.4 | 47.35 | 63.16 | 124.65 | 28.01 | 6.78 | 34.79 | 20.97 | 49.59 | 21.89 | 3.36 |
| 86 | ICGV 14224 | 51 | 17.1 | 10.3 | 49.66 | 71.93 | 129.14 | 26.48 | 10.46 | 36.94 | 26.77 | 65.74 | 28.27 | 6.88 |
| 87 | ICGV 14232 | 51.25 | 19.05 | 11.35 | 50.76 | 68.93 | 126.40 | 24.40 | 10.94 | 35.35 | 30.89 | 53.39 | 28.80 | 5.84 |
| 88 | ICGV 07262 | 52.25 | 17.2 | 8.5 | 47.01 | 85.62 | 125.87 | 22.34 | 8.96 | 31.31 | 27.94 | 64.38 | 32.34 | 5.77 |
| 89 | ICGV 07247 | 51.5 | 15.05 | 10.55 | 49.39 | 60.32 | 117.10 | 26.39 | 14.81 | 41.20 | 35.58 | 51.56 | 26.16 | 7.64 |
| 90 | ICGV 10371 | 51.25 | 18.05 | 9.5 | 47.49 | 79.83 | 128.32 | 24.93 | 8.87 | 33.80 | 25.63 | 61.09 | 25.38 | 5.42 |
| 91 | ICGV 10373 | 52 | 20 | 9.9 | 50.02 | 67.16 | 129.11 | 26.04 | 11.51 | 37.56 | 30.74 | 54.10 | 23.89 | 6.23 |
| 92 | ICGV 10379 | 52.25 | 19.55 | 9.7 | 50.21 | 79.26 | 132.60 | 29.43 | 10.90 | 40.33 | 27.04 | 65.63 | 27.17 | 7.15 |
| 93 | ICGV 15094 | 52.25 | 22.75 | 11.35 | 49.38 | 54.44 | 125.58 | 28.56 | 8.08 | 36.64 | 21.87 | 55.82 | 29.94 | 4.51 |
| 94 | ICGV 87846 | 52.25 | 24.1 | 11.5 | 46.34 | 81.84 | 122.90 | 29.66 | 11.23 | 40.89 | 27.17 | 61.55 | 37.64 | 6.91 |
| 95 | ICGV 86699 | 50 | 19.45 | 10.35 | 49.46 | 70.56 | 117.92 | 25.99 | 8.63 | 34.62 | 24.86 | 52.73 | 23.20 | 4.55 |
| 96 | GG 20 | 51 | 17.15 | 8.6 | 48.23 | 79.98 | 128.37 | 27.24 | 12.96 | 40.19 | 28.17 | 63.34 | 28.44 | 8.21 |
| 97 | ICGV 171007 | 50 | 19.9 | 9.75 | 53.33 | 62.59 | 125.56 | 24.95 | 8.64 | 33.58 | 25.02 | 56.24 | 29.62 | 4.86 |
| 98 | ICGV 171027 | 53 | 20.2 | 11 | 44.73 | 80.32 | 127.42 | 26.96 | 7.41 | 34.37 | 21.33 | 63.16 | 30.14 | 4.68 |
| 99 | ICGV 181006 | 52.25 | 22.55 | 12.4 | 48.07 | 67.79 | 131.27 | 32.78 | 8.53 | 41.31 | 20.36 | 58.45 | 24.03 | 4.98 |
| 100 | ICGV 181033 | 51.25 | 19.85 | 10.45 | 50.50 | 83.37 | 139.14 | 28.34 | 11.79 | 40.12 | 29.17 | 65.38 | 31.06 | 7.71 |
|  |  |  |  |  |  |  |  |  |  |  |  |  |  |  |
|  | |  |  |  |  |  |  |  |  |  |  |  |  |  |
| Table S2. Continued. | | | | | | | | | | | | | | |
|  | | DF | PH | PB | SCMR | LRWC | SLA | HAULM | POD | TBM | HI | SHP | HSW | SY |
| CV (%) | | 2.47 | 15 | 23.77 | 7.97 | 11.85 | 11.9 | 20.45 | 26.66 | 17.44 | 19.43 | 5.96 | 11.08 | 30.3 |
| SE | | 1.25 | 2.81 | 2.11 | 3.82 | 8.19 | 15.53 | 5.18 | 2.87 | 5.29 | 7.02 | 3.35 | 2.98 | 1.82 |
| LSD (5%) | | 1.74 | 3.92 | 2.94 | 5.33 | 11.44 | 21.24 | 7.24 | 4.01 | 7.39 | 9.8 | 4.68 | 4.17 | 2.54 |

DF=days to 50% flowering, PH=plant height, PB=number of primary branches per plant, SCMR=SPAD chlorophyll meter reading, LRWC=leaf relative water content, SLA=specific leaf area (cm^2^ g^-1^), HAULM=haulm weight (g plant^-1^), SHP=shelling percentage, HSW=hundred seed weight(g), PY=pod yield (g plant^-1^ ), HI=harvest index (%), TBM=total biomass production ( g plant^-1^ ) (g), SY=seed (g plant^-1^).
