# Supplementary material for: Assessment of the genetic diversity and population structure of groundnut germplasm collections using phenotypic traits and SNP markers: Implications for drought tolerance breeding
Source: PLoS One. 2021 Nov 17;16(11):e0259883. doi: 10.1371/journal.pone.0259883 (PMC8598071; doi:10.1371/journal.pone.0259883)
Supplement: S5 Table — (DOCX) [file pone.0259883.s005.docx]

S5 Table. Inferred ancestry of individuals and degree of admixture among 99 groundnut genotypes.

| Sr. No. | Genotype | Market type | Inferred ancestry of individuals | | | Inferred cluster |
| --- | --- | --- | --- | --- | --- | --- |
| 1 | ICGV 94118 | Spanish bunch | 0.194 | 0.806 | 0 | CL2 |
| 2 | ICGV 11422 | Spanish bunch | 0.925 | 0.075 | 0 | CL1 |
| 3 | ICGV 06040 | Spanish bunch | 0.502 | 0 | 0.498 | ADMIX |
| 4 | ICGV 10373 | Virginia bunch | 0.57 | 0.362 | 0.068 | ADMIX |
| 5 | ICGV 13254 | Spanish bunch | 0.16 | 0.84 | 0 | CL2 |
| 6 | ICGV 99241 | Spanish bunch | 0.257 | 0.598 | 0.144 | ADMIX |
| 7 | ICGV 181489 | Spanish bunch | 0.245 | 0.346 | 0.409 | ADMIX |
| 8 | ICGV 13219 | Spanish bunch | 0.002 | 0.131 | 0.867 | CL3 |
| 9 | ICGV 171013 | Spanish bunch | 0.001 | 0 | 0.999 | CL3 |
| 10 | ICGV 14030 | Spanish bunch | 0.926 | 0.001 | 0.073 | CL1 |
| 11 | ICGV 91223 | Spanish bunch | 0.073 | 0.833 | 0.094 | CL2 |
| 12 | ICGV 16688 | Spanish bunch | 0.372 | 0.627 | 0 | ADMIX |
| 13 | ICGV 00187 | Spanish bunch | 0.163 | 0.837 | 0 | CL2 |
| 14 | ICGV 06146 | Spanish bunch | 1 | 0 | 0 | CL1 |
| 15 | ICGV 01265 | Spanish bunch | 0.001 | 0 | 0.999 | CL3 |
| 16 | ICGV16686 | Spanish bunch | 0.294 | 0.705 | 0 | CL2 |
| 17 | ICGV 15073 | Spanish bunch | 0.352 | 0.648 | 0 | ADMIX |
| 18 | ICGV 97087 | Spanish bunch | 0.002 | 0.973 | 0.026 | CL2 |
| 19 | ICGV 13200 | Spanish bunch | 0.964 | 0 | 0.036 | CL1 |
| 20 | ICGV 01279 | Spanish bunch | 0.983 | 0.003 | 0.015 | CL1 |
| 21 | ICGV 15074 | Spanish bunch | 0.344 | 0.656 | 0 | ADMIX |
| 22 | ICGV 181063 | Spanish bunch | 0.024 | 0.701 | 0.275 | CL2 |
| 23 | ICGV 10178 | Spanish bunch | 0.717 | 0.283 | 0 | CL1 |
| 24 | ICGV 11418 | Spanish bunch | 0.928 | 0.072 | 0 | CL1 |
| 25 | ICGV 86031 | Spanish bunch | 0.002 | 0 | 0.998 | CL3 |
| 26 | ICGV 07222 | Spanish bunch | 1 | 0 | 0 | CL1 |
| 27 | ICGV 93260 | Spanish bunch | 0.001 | 0 | 0.999 | CL3 |
| 28 | ICGV 98412 | Spanish bunch | 0.001 | 0 | 0.998 | CL3 |
| 29 | ICGV 93261 | Spanish bunch | 0.001 | 0 | 0.999 | CL3 |
| 30 | ICGV 07220 | Spanish bunch | 1 | 0 | 0 | CL1 |
| 31 | ICGV 07010 | Spanish bunch | 0.254 | 0.015 | 0.731 | CL3 |
| 32 | ICGV 171046 | Spanish bunch | 0.92 | 0.001 | 0.079 | CL1 |
| 33 | ICGV 92121 | Spanish bunch | 0.012 | 0 | 0.988 | CL3 |
| 34 | ICGV 13207 | Spanish bunch | 0.012 | 0.236 | 0.752 | CL3 |
| 35 | ICGV 171026 | Spanish bunch | 0.721 | 0.001 | 0.278 | CL1 |
| 36 | ICGV 93128 | Spanish bunch | 0.106 | 0 | 0.894 | CL3 |
| 37 | ICGV 15019 | Spanish bunch | 0.209 | 0.006 | 0.785 | CL3 |
| 38 | ICGV 00350 | Spanish bunch | 0.119 | 0.702 | 0.179 | CL2 |
| 39 | ICGV 01260 | Spanish bunch | 0.2 | 0 | 0.799 | CL3 |
| 40 | ICGV 13317 | Spanish bunch | 0.547 | 0 | 0.453 | ADMIX |
| 41 | ICGV 02266 | Spanish bunch | 0.232 | 0.333 | 0.435 | ADMIX |
| 42 | ICGV 07120 | Spanish bunch | 0.001 | 0.948 | 0.051 | CL2 |
| 43 | ICGV 00351 | Spanish bunch | 0.184 | 0.567 | 0.249 | ADMIX |
| 44 | ICGV 181017 | Spanish bunch | 0.906 | 0 | 0.094 | CL1 |
| 45 | ICGV 96174 | Spanish bunch | 0.001 | 0.995 | 0.004 | CL2 |
| 46 | ICGV 10143 | Spanish bunch | 0.997 | 0.002 | 0.001 | CL1 |
| 47 | ICGV 181026 | Spanish bunch | 0.868 | 0 | 0.132 | CL1 |
| Table S5. Continued. | | | | | | |
| Sr. No. | Genotypes | Market type | Inferred ancestry of individuals | | | Inferred cluster |
| 48 | ICGV 14001 | Spanish bunch | 1 | 0 | 0 | CL1 |
| 49 | GPBD 4 | Spanish bunch | 0.023 | 0.393 | 0.585 | ADMIX |
| 50 | ICGV 98077 | Spanish bunch | 0.667 | 0.257 | 0.076 | ADMIX |
| 51 | ICGV 16667 | Spanish bunch | 0.328 | 0.672 | 0 | ADMIX |
| 52 | ICGV 15083 | Spanish bunch | 0.432 | 0.568 | 0 | ADMIX |
| 53 | ICGV 00162 | Spanish bunch | 0.13 | 0.869 | 0 | CL2 |
| 54 | ICGV 00211 | Spanish bunch | 0.597 | 0.401 | 0.002 | ADMIX |
| 55 | ICGV 11380 | Spanish bunch | 0.906 | 0.003 | 0.091 | CL1 |
| 56 | ICGV 14421 | Spanish bunch | 0.059 | 0.25 | 0.692 | CL3 |
| 57 | ICGV 16005 | Spanish bunch | 0.292 | 0.415 | 0.293 | ADMIX |
| 58 | ICGV 95066 | Spanish bunch | 0.031 | 0 | 0.969 | CL3 |
| 59 | ICGV 86590 | Spanish bunch | 0.003 | 0.001 | 0.996 | CL3 |
| 60 | ICGV 171039 | Spanish bunch | 0.348 | 0.553 | 0.099 | ADMIX |
| 61 | ICGV 99019 | Spanish bunch | 0.122 | 0.878 | 0 | CL2 |
| 62 | ICGV 11396 | Spanish bunch | 1 | 0 | 0 | CL1 |
| 63 | ICGV 181490 | Spanish bunch | 0.004 | 0 | 0.996 | CL2 |
| 64 | ICGV 86015 | Spanish bunch | 0.001 | 0.4 | 0.599 | ADMIX |
| 65 | ICGV 13189 | Spanish bunch | 0.006 | 0.034 | 0.959 | CL3 |
| 66 | ICGV 00213 | Spanish bunch | 0.091 | 0.909 | 0 | CL2 |
| 67 | ICGV 06039 | Spanish bunch | 0.545 | 0 | 0.454 | ADMIX |
| 68 | ICGV 03043 | Spanish bunch | 1 | 0 | 0 | CL1 |
| 69 | ICGV 14224 | Virginia bunch | 0.37 | 0.323 | 0.308 | ADMIX |
| 70 | ICGV 171007 | Virginia bunch | 0.088 | 0.001 | 0.912 | CL3 |
| 71 | ICGV 171027 | Virginia bunch | 0.402 | 0 | 0.597 | ADMIX |
| 72 | ICGV 10379 | Virginia bunch | 0.48 | 0.52 | 0 | ADMIX |
| 73 | ICGV 181006 | Virginia bunch | 0.523 | 0.374 | 0.103 | ADMIX |
| 74 | ICGV 181033 | Virginia bunch | 0.893 | 0 | 0.107 | CL1 |
| 75 | ICGV 06175 | Virginia bunch | 0.116 | 0.884 | 0 | CL2 |
| 76 | ICGV 96165 | Virginia bunch | 0.44 | 0.56 | 0 | ADMIX |
| 77 | ICGV 00246 | Virginia bunch | 0.116 | 0.884 | 0 | CL2 |
| 78 | ICGV 00064 | Virginia bunch | 0.129 | 0.798 | 0.073 | CL2 |
| 79 | ICGV 07247 | Virginia bunch | 1 | 0 | 0 | CL1 |
| 80 | ICGV 97150 | Virginia bunch | 0.001 | 0.999 | 0 | CL2 |
| 81 | ICGV 87846 | Virginia bunch | 0.072 | 0.928 | 0 | CL2 |
| 82 | ICGV 03287 | Virginia bunch | 1 | 0 | 0 | CL1 |
| 83 | ICGV 07262 | Virginia bunch | 1 | 0 | 0 | CL2 |
| 84 | GG20 | Virginia bunch | 0.136 | 0.022 | 0.842 | CL3 |
| 85 | ICGV 95111 | Virginia bunch | 0.392 | 0.306 | 0.302 | ADMIX |
| 86 | ICGV 96266 | Virginia bunch | 0.009 | 0.606 | 0.384 | ADMIX |
| 87 | ICGV 97115 | Virginia bunch | 0.06 | 0.275 | 0.665 | ADMIX |
| 88 | ICGV 10371 | Virginia bunch | 0.572 | 0.362 | 0.066 | ADMIX |
| 89 | ICGV 93162 | Virginia bunch | 0.001 | 0.072 | 0.927 | CL3 |
| 90 | ICGV 98385 | Virginia bunch | 0.001 | 0.999 | 0 | CL2 |
| 91 | ICGV 14232 | Virginia bunch | 0.36 | 0.332 | 0.309 | ADMIX |
| 92 | ICGV 05057 | Virginia bunch | 0.436 | 0.21 | 0.355 | ADMIX |
| 93 | ICGV 92054 | Virginia bunch | 0.146 | 0.18 | 0.674 | ADMIX |
| 94 | ICGV 15094 | Virginia bunch | 0.641 | 0.3 | 0.058 | ADMIX |
| 95 | ICGV 98184 | Virginia bunch | 1 | 0 | 0 | CL1 |
| 96 | ICGV 86699 | Virginia bunch | 0.001 | 0.999 | 0 | CL2 |
| Table S5. Continued. | | | | | | |
| Sr. No. | Genotypes | Market type | Inferred ancestry of individuals | | | Inferred cluster |
| 97 | ICGV 01491 | Virginia bunch | 0.003 | 0 | 0.997 | CL3 |
| 98 | ICGV 05155 | Spanish bunch | 0.83 | 0.17 | 0 | CL1 |
| 99 | ICGV 03042 | Spanish bunch | 0.999 | 0.001 | 0 | CL1 |

CL=cluster, ADMIX=admixture
